# In Vivo Reinsertion of Excised Episomes by the V(D)J Recombinase: A Potential Threat to Genomic Stability

**DOI:** 10.1371/journal.pbio.0050043

**Published:** 2007-02-13

**Authors:** Katrina Vanura, Bertrand Montpellier, Trang Le, Salvatore Spicuglia, Jean-Marc Navarro, Olivier Cabaud, Sandrine Roulland, Elodie Vachez, Immo Prinz, Pierre Ferrier, Rodrig Marculescu, Ulrich Jäger, Bertrand Nadel

**Affiliations:** 1 Department of Internal Medicine I, Division of Hematology, Medical University of Vienna, Vienna, Austria; 2 Centre d'Immunologie de Marseille-Luminy, Université de la Méditerranée, Marseille, France; 3 Institut National de la Santé et de la Recherche Médicale U631, Marseille, France; 4 Centre National de la Recherche Scientifique UMR6102, Marseille, France; Scripps Research Institute, United States of America

## Abstract

It has long been thought that signal joints, the byproducts of V(D)J recombination, are not involved in the dynamics of the rearrangement process. Evidence has now started to accumulate that this is not the case, and that signal joints play unsuspected roles in events that might compromise genomic integrity. Here we show both ex vivo and in vivo that the episomal circles excised during the normal process of receptor gene rearrangement may be reintegrated into the genome through *trans*-V(D)J recombination occurring between the episomal signal joint and an immunoglobulin/T-cell receptor target. We further demonstrate that cryptic recombination sites involved in T-cell acute lymphoblastic leukemia–associated chromosomal translocations constitute hotspots of insertion. Eventually, the identification of two in vivo cases associating episomal reintegration and chromosomal translocation suggests that reintegration events are linked to genomic instability. Altogether, our data suggest that V(D)J-mediated reintegration of episomal circles, an event likely eluding classical cytogenetic screenings, might represent an additional potent source of genomic instability and lymphoid cancer.

## Author Summary

Lymphoid cells recognize billions of pathogens as a result of gene rearrangements that generate pathogen-specific B- and T-cell receptors. This genetic reshuffling, called V(D)J recombination, occasionally misfires and damages genomic integrity. When such aberrations dysregulate proto-oncogenes, cancer ensues. It has become increasingly clear that multiple oncogenes acting in different cellular pathways can cooperate to cause cancer. Nevertheless, in the case of T-cell acute lymphoblastic leukemia, about a third of cases display oncogene activation in the absence of identified aberration, suggesting the presence of additional mechanisms of chromosomal alteration. In the hunt for such mechanisms, episomal circles (DNA segments that are excised during V(D)J recombination) have recently drawn attention. Moreover, signal joints, short sequences formed after gene rearrangements, once considered harmless, now appear to take part in events that might compromise genomic integrity. Using ex vivo recombination assays and genetically modified mice, we demonstrate that episomal circles may be reintegrated into the genome through recombination occurring between the episomal signal joints and a T-cell receptor target. Furthermore, we show that cryptic recombination sites located in the vicinity of oncogenes constitute hotspots of episomal insertion. Altogether, our results suggest that reintegration of excised episomal circles constitute a potential source of genomic instability and cancer in leukemia and lymphoma.

## Abstract 3

Episomal DNA circles are the by-products of immunoreceptor gene rearrangements in lymphoid cells. Episomal circles can be reintegrated into the genome by *trans*-V(D)J recombination and cause oncogene deregulation.

## Introduction

**Figure 1:**
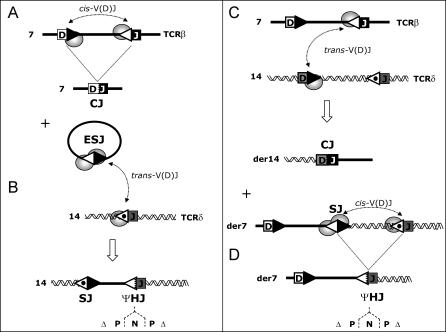
Two Models of Illegitimate V(D)J Recombination (A) V(D)J recombination generating an ESJ. (B) Ongoing V(D)J recombination of the ESJ with a RSS target in *trans,* leading to reintegration of the excised EC in the genome. The specific *trans*-CSJ and ΨHJ breakpoint signatures of the episomal reintegration are depicted. (C) V(D)J-mediated translocation generating one *trans*-chromosomal CJ and one *trans*-CSJ. (D) Ongoing V(D)J recombination of a CSJ in *cis.* The illustration depicts a CSJ generated through V(D)J-mediated translocation, but the same principle stands for a CSJ generated through V(D)J recombination by inversion. White triangles, 12-RSS; black triangles, 23-RSS; dotted white triangles, IG/TCR or cryptic 12-RSS; rectangles, IG/TCR gene segment or oncogene activated through rearrangement; ellipses, RAG-1/2. Indicative chromosomes and derivative chromosomes are shown. Dented line in the ΨHJ and CJ represents processing at the junction (Δ, nucleotide deletion; P, P nucleotide addition; N, N nucleotide addition).

V(D)J recombination is a unique mechanism of somatic recombination aimed to provide a large antigen receptor repertoire in T and B cells (for review, see [1] and references therein). During this process, the variable (V) diversity (D) and joining (J) gene segments present within the immunoglobulin (IG) and T-cell receptor (TCR) loci, are assembled to form a complete VDJ exon encoding the variable region of the IG/TCR (Figure 1A). The recombination requires the presence of specific motifs flanking all the V, D, and J gene segments (12– and 23–recombination signal sequences [RSSs]), and allowing the recruitment, binding, and proper positioning of the products of the recombination activating genes 1 and 2 *(RAG-1/2).* Recent data suggest that in vivo, RAG-1/2 proteins initiate the rearrangement by performing a first single-strand nick at the exact border between a 12-RSS and its adjacent coding gene segment [2]. This leads to the capture of a 23-RSS, the formation of a 12/23 synaptic complex in which the two DNA/protein structures are held in close juxtaposition, and the generation of another nick at the captured 23-RSS. Within this complex, RAG-1/2 catalyzes a *trans*-esterification reaction in which each liberated hydroxyl group attacks the opposite DNA strand [3]; this generates four broken ends held in a postcleavage synaptic complex: two blunt RSSs or signal ends (SEs), and two covalently sealed hairpin coding ends (CEs). The broken ends are then efficiently repaired by the nonhomologous end-joining (NHEJ) pathway [4–6]; on the one hand, hairpins present at the CEs are resolved through the Artemis endonuclease activity; when opened at bases off the apex, hairpin opening generates overhanging flaps, which, if filled in by a DNA polymerase activity, form palindromic (P) stretches. Nontemplated (N) nucleotides may be added de novo by the terminal deoxynucleotidyl transferase (TdT), and/or nucleotides may be deleted from the CEs. Ligation of the processed CEs forms a highly diversified coding joint (CJ). By contrast, the two SEs present in the synaptic complex undergo only limited processing (some N addition and rare nucleotide deletion) before joining into signal joints (SJs). While CJs give rise to the functional recombination products, the SJs are merely the byproduct of V(D)J recombination.

SJs have until recently been assumed to be “harmless” and irrelevant in the dynamics of the V(D)J recombination process, but evidence now starts to accumulate that this is not the case, and that they play unsuspected roles in events which might compromise genomic integrity [7,8]. SJs are indeed constituted of two functional RSSs fused back to back, each of which therefore potentially capable of further V(D)J rearrangement in presence of RAG-1/2. The issue of SJ reactivity was initially addressed ex vivo by the use of integrated minilocus and transient extrachromosomal recombination substrates containing germline gene segments flanked by their RSSs, and undergoing rearrangement in culture [9–11]. Both integrative and extrachromosomal experiments indicated that, following a first rearrangement by inversion, the SJ produced was indeed reactive, and could engage into further cycles of rearrangement with RSS partners in *cis* (similar to Figure 1C and 1D). In vivo and ex vivo observations have revealed that the products resulting from such secondary SJ rearrangements consist of one new SJ and one hybrid RSS/coding-segment junction (hybrid joint [HJ]), albeit with the molecular features of a CJ (i.e., with N nucleotide insertion, and extensive nucleotide deletion and P nucleotide addition at both the RSS and coding segment sides; Figure 1D) [8–10,12]. This junction, which we refer to as a “pseudo-hybrid” joint (ΨHJ), is thereby morphologically distinguishable from CJs, SJs, and to a large extent from “genuine” HJs [13–18]. ΨHJs constitute therefore specific signatures of such ongoing SJ rearrangement events. Interestingly, recent in vivo data suggest that IGK/IGL rearrangement hierarchy and isotypic exclusion might in part be achieved by ongoing SJ recombination [12]. Thus, SJ reactivity might have also evolved as part of the dynamics of the V(D)J rearrangement process. Eventually, the pathological counterpart of this possible physiological extension of the V(D)J recombination capability has also been shown to occur in cases of oncogenic chromosomal translocation, in which ongoing rearrangement of the resulting chromosomal SJ (CSJ) constitutes the source of oncogene activation [8].

In the normal process of V(D)J recombination, the large majority of SJs produced is however not retained on the chromosome, but excised on episomal circles (ECs; Figure 1A). Because ex vivo RAG binding (or rebinding) also efficiently takes place on episomal SJs (ESJs), leading to SJ recleavage and, at least in vitro, to RAG transposition [7], we reasoned that ongoing *trans*-V(D)J recombination might also and concurrently occur (Figure 1B). This might result in the same type of *insertion* of the whole circle into the genome as previously observed in vivo for RAG-mediated transposition [19], with the important difference that it would in this case employ *trans*-V(D)J recombination [20–23], a process potentially more efficient than RAG transposition [15,24–28]. Both mechanisms might obviously lead to similar genomic instability events, including oncogenic activation/deregulation. In this report, we investigated V(D)J-mediated ESJ insertion as an additional potent source of genomic instability and oncogenic deregulation in lymphoid cells.

## Results

### ESJs Undergo *trans*-V(D)J Recombination Ex Vivo

**Figure 2:**
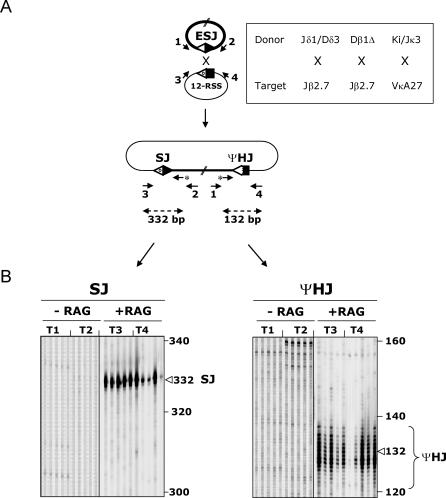
Ex Vivo *trans*-V(D)J Recombination Assay (A) Schematic representation of *trans*-V(D)J recombination between an ESJ (donor) and a 12-RSS (target). The expected breakpoints (one SJ and one ΨHJ) are shown. The various donor/target combinations assayed are boxed. PCR primers are depicted by arrows. Nested IR800-labeled PE primers are indicated by an asterisk. (B) Typical example of PCR/PE assays obtained from the (Dβ1Δ) ESJ × Jβ2.7 12-RSS combination. The expected sizes of the PE products are indicated. As the resolution of the PE assay is to the base, ΨHJ patterns typically show multiple bands (corresponding to the spectra of nucleotide addition and deletion), while SJ patterns are typically centered on a major band (corresponding to no or limited nucleotide processing). PE assays shown were performed on 0.75 μl PCR. Five independent PCRs from two independent transfections performed with (T3–T4) or without RAG-1/2 (T1–T2) are shown for each junction.

To investigate the possibility that excised episomes can reintegrate the genome through ongoing recombination of the SJ, we first assessed the ability of an ESJ to undergo *trans*-V(D)J recombination in an ex vivo *trans*-recombination substrate assay [20] (Figure 2A). Three different human ESJs and two standard RSSs were cloned in separate extrachromosomal recombination substrates; two genuine SJs were used as “donor” ESJ plasmids: Jδ1/Dδ3 and Ki/Jκ3 [12]; furthermore, an artificial Dβ1Δ ESJ was generated by mutagenesis deletion of the 12-bp Dβ1 coding sequence located between the Dβ1 5′ and 3′ RSSs; the human Jβ2.7 and VκA27 gene segments were used as 12-RSS “target” substrates. NIH3T3 fibroblasts were cotransfected with donor and target plasmids either with or without RAG-1/2 and TdT expression vectors. Bulk plasmid DNA was recovered after 48 h of culture, and junctions resulting from *trans*-recombination between the ESJ and the target RSS were amplified in a single round PCR. Primer combinations were designed to detect the 2 expected integration breakpoints complying with a 12/23 synapsis: combination (3 + 2) was used to detect putative SJs, and combination (1 + 4) was used to detect putative ΨHJs (Figure 2A). PCR products were revealed by an IRD800-labeled primer extension (PE) assay allowing a precise to-the-base resolution of the amplified species (see Materials and Methods).

In absence of the RAGs, faint and nonrecurrent PCR products scattered at various sizes were obtained for both primer combinations (e.g., Figure 2B, ΨHJ T2, ~160 bp; additional bands are also present outside the visualized parts of the gels). To assess the identity of such junctions, double-nested secondary PCR were performed, and the amplification products were cloned and sequenced. Sequence analysis confirmed the occurrence of junctions that were not generated by RAG-mediated recombination, but rather due to random breaks joined through NHEJs and/or homologous recombination pathways (not shown). Such junctions, collectively referred to as “break/repair,” have been previously described, and represent the RAG-independent recombination background of the *trans*-V(D)J assay [29].

**Figure 3:**
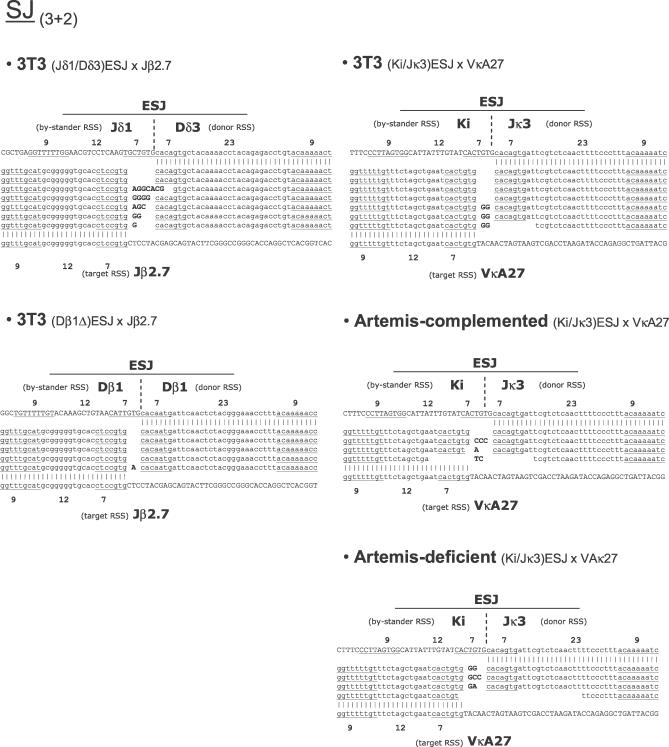
Breakpoint Sequences of Ex Vivo *trans*-V(D)J Recombinants SJ (primer combination 3 + 2). Top and bottom lanes depict sequences before recombination (heptamers, spacers and nonamers are specified). Recombined clones are depicted between top and bottom lanes, with homology to the unrecombined sequences indicated by vertical lines. Lower cases represent reactive RSS involved in the *trans*-V(D)J recombination reactions (specified as donor RSS and target RSS). Upper cases represent coding segments, or bystander RSSs in the ESJ behaving like coding segments (specified as bystander RSSs). Italics indicate potential P nucleotides; bold type, N nucleotides; heptamers and nonamers are underlined; identical sequences shown on separate lanes are issued from distinct transfections and represent therefore independent junctions. Since identical sequences were often obtained in a given transfection, this representation might in some cases bias the representation towards processed junctions (because they acquire distinguishing features).

**Figure 4:**
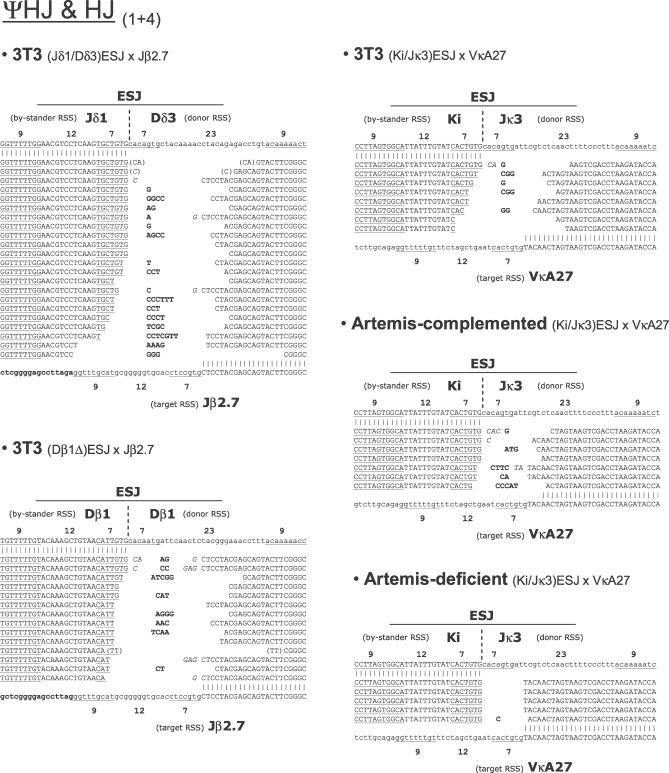
Breakpoint Sequences of Ex Vivo *trans*-V(D)J Recombinants ΨHJ and/or HJ (primer combination 1 + 4). Nucleotides in parenthesis are ambiguous and could be assigned to either side of the joint. See also legend to Figure 3.

In presence of RAGs, however, more intense and recurrent PCR products of the expected sizes were obtained for both PCR combinations (typical examples are illustrated for the Dβ1Δ × Jβ2.7 combination in Figure 2B). SJ primer combination (3 + 2) usually displayed one major band and few minor species around that position, as expected from a standard SJ, which generally presents limited nucleotide processing. ΨHJ primer combination (1 + 4), on the other hand, displayed a pattern of intense bands around the expected size, representing the typical spectra of largely processed junctions. Sequence analysis of cloned products from double-nested secondary PCR confirmed the presence of the two specific breakpoints expected from V(D)J-mediated ongoing ESJ recombination in most cases (Figures 3 and 4; also, see below). Thus, and as anticipated from previous studies describing ongoing recombination of SJ with targets in *cis,* our results demonstrate that ESJs are also capable of ongoing efficient RAG-mediated recombination with RSS targets in *trans* in the context of a 12/23 synapsis. However, as the ESJ is formed by a functional 12-RSS and a functional 23-RSS, both potentially able to bind the RAGs, we next wondered if this particular structure might allow to bypass the 12/23 rule for synapsis and give rise to additional recombination products that we would fail to detect with the two primer combinations used above. Double-nested PCR with the two complementary primer combinations (1 + 3) and (2 + 4) (Figure 2A) corresponding to a 12/12 synapsis were thus performed on the same bulk DNA. Such combinations, however, gave rise to only weak amplification products. Cloning and sequencing confirmed in most cases the occurrence of the symmetrical 12/12 SJ (1 + 3) and ΨHJ (2 + 4) (Figure S1A). This suggests that although a fraction of the *trans*-recombination can occur in violation of the 12/23 rule (as in normal V(D)J recombination), this represents a minor population, and the large majority of the RAG-mediated recombinants are detected with primer combinations (3 + 2) and (1 + 4), in accordance with a 12/23 synapsis between the ESJ and its RSS target. Accordingly, the use of ESJ donors made of two 12-RSS in the context of a 12-RSS target did not give rise to any specific amplified signal, while each of the two RSS from an ESJ donor made of two 23-RSS gave rise to efficient recombination in the context of the same 12-RSS target (Figure S1B). In addition, the use of ESJ donor constructs made of one functional and one nonamerless RSS through deletional mutagenesis showed that while deletion of the nonamer from the reactive 23-RSS completely abolished recombination, deletion of the nonamer from the bystander 12-RSS did not modify the overall efficiency (Figure S1C). Altogether, our data clearly indicate that *trans*-V(D)J recombination of ESJ obeys the 12/23 rule and is not dependent on alternative mechanisms such as ESJ opening by nick–nick (see below for a potential role of nick–nick in the generation of some junctions).

### *trans*-V(D)J Recombination Is the Major Pathway of RAG-Mediated ESJ Recombination

We next analyzed in details the sequences issued from recombination of the ESJ (Figure 3 and 4). SJs obtained were morphologically undistinguishable from standard SJs, with the presence of some N insertion and limited nucleotide deletion (Figure 3; 3T3). Likewise, detailed analysis of the ΨHJ revealed that in most sequences, end processing similar to that of a standard CJ (nucleotide deletion, N and P nucleotide addition) occurred at both the SE (the bystander 12-RSS of the ESJ) and the CE sides of the joint (Figure 4; 3T3). This strongly suggests that once engaged in synapsis, and despite its ability to bind the RAGs, the bystander RSS of the ESJ mostly behaves as a coding segment, and undergoes therefore the hairpin formation step, followed by hairpin resolution and further processing (Figure S2, left panel). Nevertheless, the presence of sequences containing a full-size ESJ bystander RSS (e.g., Jδ1; Figure 4) together with the ambiguity in the assignment of P nucleotides in presence of TdT, suggested possible RAG binding on both ESJ RSSs, and involvement of alternative pathways of RAG-mediated recombination. To confirm the involvement of *trans*-V(D)J recombination and to test the potential contribution of alternative pathways, the ESJ assay was repeated in the GUETEL Artemis-deficient cell line [30]. During standard V(D)J recombination, failure to resolve hairpin CEs in absence of the Artemis endonuclease results in the absence of CJ formation without impeding SJ formation [31–33]. Similarly, the absence of Artemis in the ESJ assay should prevent ΨHJ formation without hindering the generation of SJs. Following cotransfection of the GUETEL cell line with the (Ki/Jκ3)ESJ/VκA27 couple, PCR was performed as above and the amplification products cloned and sequenced. As expected, SJs were obtained in absence of Artemis and were undistinguishable from SJs obtained both in the Artemis-proficient 3T3 cells and in the GUETEL cell line complemented with Artemis (GUETEL-A; [30] and Figure 3). Despite the absence of Artemis, weak amplification products were also obtained for the ΨHJ PCR combination (1 + 4). However, the sequenced junctions displayed virtual absence of N insertion and nucleotide processing (Figure 4), in sharp contrast to the morphology of ΨHJs obtained in Artemis-proficient cells (3T3 and GUETEL-A). The features of the Artemis^−/−^ junctions are however strongly reminiscent of HJs generated by the “RAG-mediated joining” pathway [15–18]. RAG-mediated joining is an NHEJ-independent pathway related to RAG transposition in which a direct attack of a free 3′ hydroxyl group from the SE into the hairpinned CE bypasses the hairpin resolution step (illustrated in Figure S2, right panel); this usually results in the generation of a class of HJs displaying a full-size RSS joined to a coding sequence with limited processing (depending on the position of the attack in the hairpin). The presence of such junctions in the absence of Artemis suggests that both *trans*-V(D)J recombination and RAG-mediated joining concurrently occur to generate ΨHJs and HJs, respectively. However, the virtual absence of such junctions in Artemis-proficient cells (3T3, Artemis-complemented) strongly suggests that RAG-mediated joining constitutes a minor recombination pathway compared to *trans-*V(D)J recombination. Thus, the junctions with a full-size ESJ bystander RSS initially observed in Artemis-proficient cells are Artemis dependent, and are either ΨHJs in which processing is limited due to RAG-binding on the bystander RSS, or HJs generated through “RSS swapping” (Figure S2, middle panel).

Altogether, our results indicate therefore that standard *trans-*V(D)J recombination is the major pathway of RAG-mediated ESJs ongoing rearrangement.

### ESJs Are as Efficient as Standard RSSs to Undergo *trans*-V(D)J Recombination Ex Vivo

**Figure 5:**
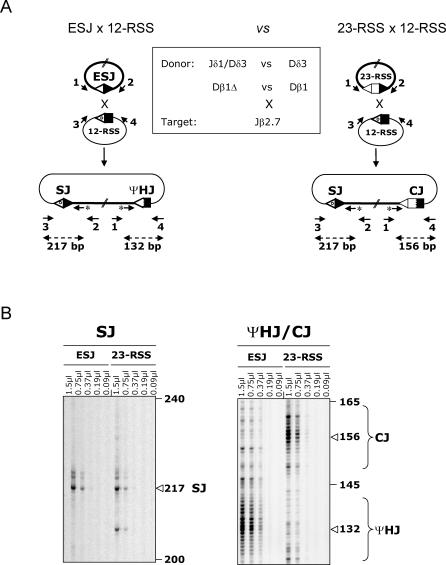
Semiquantitative Ex Vivo *trans*-V(D)J Recombination Assay (A) Schematic representation of the comparative *trans*-V(D)J recombination assay. Left panel: recombination of an ESJ 23-RSS (Jδ1/Dδ3 or Dβ1Δ) with a 12-RSS target (Jβ2.7); Right panel: recombination of a coding segment/23-RSS (Dδ3 or Dβ1) with a 12-RSS target (Jβ2.7). The expected breakpoints are shown for each case. (B) Typical example of semiquantitative PCR/PE assays obtained from the (Jδ1/Dδ3) ESJ × Jβ2.7 versus Dδ3 × Jβ2.7 comparisons. PE assays shown were performed on serial dilutions of PCR products.

How efficient is *trans*-V(D)J recombination of ESJs? In the case of recombination between two standard coding-segment RSSs, the frequency of *trans*-V(D)J recombination has been previously shown to be reduced compared to *cis*-V(D)J recombination events [20,21]. In the present case of recombination between an ESJ and its RSS target, the presence of the second RSS in the SJ could structurally impede—or on the contrary stimulate—RAG fixation and/or activity on the reactive one, and modify the overall recombination efficiency. We therefore compared in the ex vivo assay the efficiency of *trans*-V(D)J recombination of an ESJ and a RSS target with the efficiency of *trans*-V(D)J recombination between two standard coding-segment RSSs (Figure 5A). To do so, we tested two pairs of plasmids: (Jδ1/Dδ3) ESJ × Jβ2.7 versus Dδ3 × Jβ2.7, and (Dβ1ΔESJ × Jβ2.7 versus Dβ1 × Jβ2.7. Following transfection and harvesting carried out as above, semiquantitative primary PCR amplification of the breakpoints was performed, and serial dilutions were revealed by PE. As illustrated in Figure 5B for the (Jδ1/Dδ3) ESJ × Jβ2.7 versus Dδ3 × Jβ2.7 couples, no significant difference could be seen in the formation rate of SJs in the presence of an ESJ. Similarly, results showed comparable rates in the formation of a ΨHJ relative to a CJ. We conclude that ESJs are at least as efficient as standard RSSs to undergo *trans*-V(D)J recombination. Altogether, this suggests that the presence of the bystander RSS does not impede the recombination process, whether structurally (through steric constraints) or functionally (through nick–nick activity). From the mechanistic point of view, this data predicts therefore that in vivo, V(D)J-mediated reintegration of ESJs (Figure 1B) should not be different from V(D)J-mediated translocation (Figure 1C); most important, both processes should use the same RSS targets with the same efficiency.

### Cryptic RSSs Involved in Oncogenic Translocations Constitute Hotspots of Episomal Reintegration through Ongoing V(D)J Recombination of an ESJ

**Figure 6:**
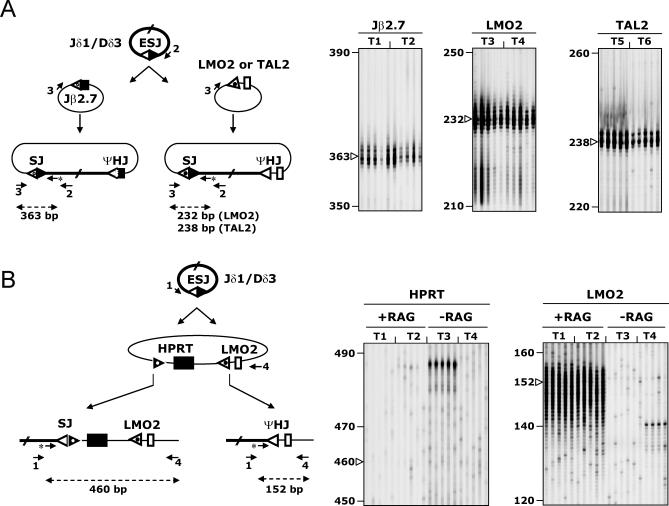
Ex Vivo SJ *trans*-V(D)J Recombination Assay with Cryptic RSS Targets (A) Recombination of (Jδ1/Dδ3) ESJ with Jβ2.7 12-RSS (white triangle with open dot), *LMO2,* or *TAL2* cryptic 12-RSS (white triangle with filled dot). PE assays shown were performed on 1 μl primary PCR. Five independent PCRs from two independent transfections (T1–T2, T3–T4, and T5–T6) are shown for each junction. Similar results were obtained when *LMO2* and Jβ2.7 RSSs were cloned on a single plasmid as competitive targets. (B) Competitive recombination of (Jδ1/Dδ3) ESJ with *HPRT* intron 1 cryptic RSS (black triangle with white dot) versus *LMO2* cryptic RSS (white triangle with filled dots). SJ, Jδ1/*HPRT*; ΨHJ, Jδ1/*LMO2.* PE assays shown were performed on 3 μl primary PCR using 30 cycles of PE. Five independent PCRs from two independent transfections performed in the presence (T1–T2) or absence of RAG-1/2 (T3–T4) are shown for each junction. Similar results were obtained when *LMO2* and *HPRT* were cloned on separate plasmids and assayed in parallel. As described in the text, absence of specific V(D)J recombination (e.g., in absence of RAG, T3 ~485 bp) favors the emergence of nonspecific and nonrecurrent recombination events (“break-repair”).

V(D)J-mediated translocations have been shown to occur not only between authentic RSSs from distinct IG/TCR loci, but also between authentic RSSs and fortuitous sequences in the genome resembling a RSS (cryptic RSSs) [34]. The mistargeting of the RAGs towards cryptic RSSs located in the vicinity of a silent proto-oncogene is a recurrent source of genomic instability and oncogenesis. Erroneous targeting of cryptic sites located near the *LMO2* and *TAL2* proto-oncogenes in t(11;14)(p13;q11) and t(7;9)(q34;q32) translocations, respectively, represent prototypical examples of such oncogenic translocations in T-cell acute lymphoblastic leukemia (T-ALL) [8,29,35–37]. Our data above suggest that in vivo, such cryptic sites might provide efficient targets for ESJ reintegration. To further define the potential oncogenic properties of episomal reintegration, we next investigated in our ex vivo assay the capacity of ESJs to target oncogenic cryptic RSS. The human *LMO2* and *TAL2* cryptic RSSs and flanking sequences were cloned in a recombination substrate plasmid (Figure 6A) and assayed in parallel to the Jβ2.7 segment as a target for the (Jδ1/Dδ3) ESJ, using the PCR/PE assay described above. Our results show a similar considerable high rate of V(D)J-mediated recombination of the ESJ with the *LMO2* and *TAL2* cryptic RSSs than with the Jβ2.7 authentic RSS (Figure 6A). To further estimate the likelihood in vivo of SJ insertion in such cryptic RSS*,* the *LMO2* versus the *HPRT* intron 1 region were assayed as competitive targets for the (Jδ1/Dδ3 ESJ (Figure 6B). The *HPRT* intron 1 region was chosen as a competitor because it contains a well-described cryptic RSS classically involved in illegitimate V(D)J-mediated deletion of exons 2–3 in vivo [38], and has also been identified as a site of ESJ reintegration in vivo ([39]; see Discussion) as well as a region of RAG transposition in vivo [19]. While recombination with the *LMO2* cryptic RSS constituted a hotspot of integration, no recombination could be observed in the *HPRT* region, neither specifically at the cryptic RSS, nor in the surrounding sequences (Figure 6B). Similar results were obtained when using a target plasmid containing a second copy of the *HPRT* fragment in a head-to-tail orientation, and forming a cruciform structure (not shown). Altogether, these data strongly suggest that cryptic sites such as *LMO2* with much higher recombinogenic potential than the *HPRT* intron 1 cryptic RSS might also be targeted in vivo by ESJ insertion and could potentially lead to oncogenic activation.

### *trans*-Chromosomal ΨHJ Breakpoints Are Readily Detectable In Vivo

We next investigated the physiological relevance of the reintegration of excised episomes through ongoing SJ recombination. As a first approach, we sought to assess if *trans*-chromosomal ΨHJ breakpoints were present and detectable in vivo, and if so, to estimate their formation rate. To do so, we designed primer combinations allowing the amplification of *trans*-TCR ΨHJ in mouse thymocyte DNA (Table 1). Additional combinations designed to amplify *trans*-TCR CJs and *trans*-TCR SJs were also performed as reference. To detect such relatively rare *trans*-TCR recombination events, we used a sensitive fluctuation PCR assay allowing the detection of less than one recombination event in a million cells (see Materials and Methods). Positive PCR-amplification replicates were obtained for all *trans*-TCR combinations in the broad range of ~1 in 1,000,000 cells to 1 in 10,000 cells. Sequence analysis of the PCR-amplification products was carried out to assess the identity of the junctions, and revealed the presence of ΨHJs (Figure S3), CJs, and SJs (not shown). Importantly, the ΨHJs obtained in vivo displayed the same molecular features as observed in the ex vivo assay, with extensive nucleotide processing on both sides of the junctions, and N/P addition. These results clearly demonstrate that ΨHJ breakpoints are indeed generated in vivo. Furthermore, such junctions are readily detectable in mouse thymocytes at a range similar to that of equivalent *trans*-CSJs and CJs. In line with our ex vivo results, this indicates that in vivo, SJs undergo efficient ongoing *cis*- and/or *trans*-rearrangement with RSS targets in the genome with surprisingly high frequency. This suggests further that in presence of RAGs, CSJs and/or ESJs are indeed very recombinogenic structures.

**Table 1:**
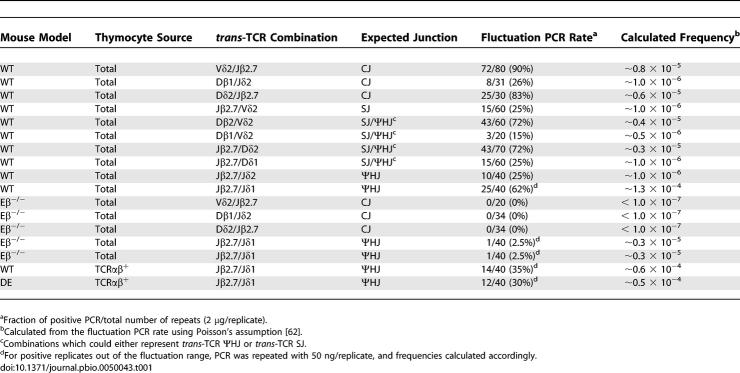
In Vivo Rates of *trans*-TCR Junctions

### Efficient In Vivo Reintegration of ECs through Ongoing V(D)J Recombination of a SJ

**Figure 7:**
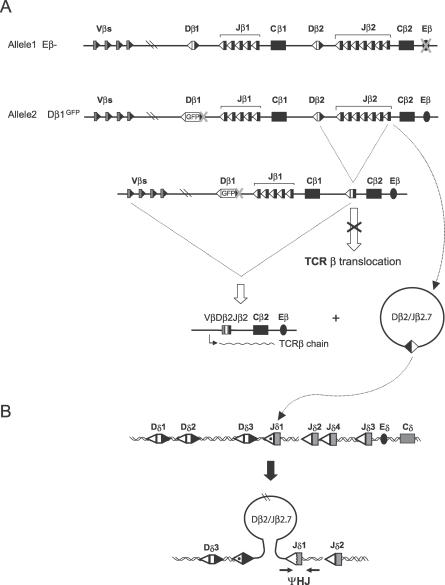
Strategy for In Vivo Detection of Episomal Reintegration in the (Dβ1^GFP^ × Eβ^−^) double-mutant (DE) mouse (A) TCRβ allelic configuration of the DE mouse. The Eβ knockout prevents efficient rearrangements on allele 1, and the GFP knockin in Dβ1 prevents D-J cluster 1 rearrangements on allele 2. In this configuration, all productive TCRβ chains are issued from Vβ rearrangements to D-J cluster 2 of allele 2. Consequently, TCRβ chain production and translocation to the TCRβ locus are mutually exclusive. The generation of a TCR excision circle carrying a (Jβ2/Dβ2)ESJ through Dβ2-Jβ2 rearrangements from the Dβ1^GFP^ allele is depicted. (B) Reintegration of (Jβ2/Dβ2) ESJs in Jδ1. In TCRαβ^+^ cell-sorted thymocytes from DE mice, the presence of a Jβ2.7/Jδ1 ΨHJ specifically signs the reintegration of (Jβ2/Dβ2)ESJ in Jδ1, and is assayed by fluctuation PCR.

Although we demonstrated above that *trans*-chromosomal ΨHJs are readily detectable in vivo, such breakpoints do not represent exclusive signatures of ESJ reintegration, as they may also derive from ongoing recombination of *trans*-CSJs with neighboring gene segments in *cis* (compare Figure 1B and 1D). To ensure that the ΨHJs observed above did not exclusively derive from CSJs issued from *trans*-TCR translocations, and to estimate the rate of episomal reintegration in vivo, we generated double mutant mice in which the generation of *trans*-CSJs is abolished (Figure 7). Eβ^−/−^ knockout mice, in which deletion of the 560-bp Eβ core generates an >100-fold reduction in TCRβ rearrangements [40] and TCRδ/β translocations (Table 1), were crossed with a Dβ1GFP knockin mouse, in which the introduction of the GFP in the Dβ1 gene segment and flanking 23-RSS abolishes Dβ1-Jβ1/2 rearrangements (SS, OC, PF, unpublished data). In the (Dβ1^GFP^ × Eβ^−^) double-mutant (DE) mice, all TCRβ chains are consequently produced via a Vβ-Dβ2-Jβ2 rearrangement from the Dβ1^GFP^ allele (unpublished data), and all TCR excision circles issued from Dβ-Jβ rearrangements carry a (Dβ2Jβ2) ESJ (Figure 7A). Because one allele is knocked out for Eβ, and the other produces a TCRβ chain, translocations to TCRβ cannot be present in TCRαβ^+^ cells from DE mice. In absence of translocation to TCRβ, no *trans*-TCRβδ CSJ is produced, and all Jβ2.7/Jδ1 ΨHJs are consequently issued from episomal reintegration. As shown in Table 1, while the rate of Jβ2.7/Jδ1 ΨHJs was decreased over ~40-fold in total thymocytes from the Eβ^−/−^ mice compared to WT, the rate of Jβ2.7/Jδ1 ΨHJs was only decreased 1.2-fold in sorted TCRαβ^+^ cells from DE mice compared to WT.

Altogether, and in agreement with the ex vivo data, these results clearly demonstrate that V(D)J-mediated episomal reintegration is indeed occurring in vivo, and at a rate comparable to that of V(D)J-mediated chromosomal translocation.

### High Rates of Chromosomal Aberrations Combining Insertion and Translocation

**Figure 8:**
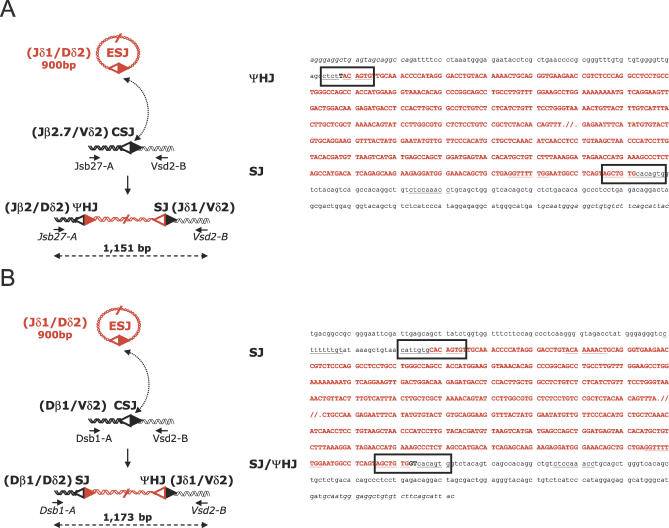
Two In Vivo Cases of Combined Chromosomal Translocation and Reintegration of an Excised EC The insertion of a (Jδ1/Dδ2) ESJ (red, upper cases) into (A) a Jβ2.7/Vδ2 *trans*-TCR or (B) a Dβ1/Vδ2 *trans*-TCR CSJ target (black, lower cases) is depicted; alternatively, ESJ reintegration could have occurred first, and provided a *trans*-TCR structure and/or breakpoints prone to V(D)J-mediated translocation. Breakpoint junctions are boxed. Black bold upper case type: N nucleotides; heptamers and nonamers are underlined. PCR primers are indicated, and shown in italics in the sequence.

During our screen of *trans*-TCR SJs described above, we noticed the presence of amplification products larger than expected. Out of a total of 310 PCR replicates of seven distinct *trans*-TCRδ/β SJ combinations, 120 were PCR positive and four were of unexpected larger size (unpublished data). Sequencing of the cloned products revealed that large amplicons resulted from *cis*-V(D)J recombination to a cryptic site in one case, and to a downstream gene segment in another case; eventually, two of the four cases showed the insertion of a sequence from the TCRδ locus with features compatible with RAG-mediated ESJ reintegration. In one of these two cases, a ~900-bp Jδ1-Dδ2 fragment was inserted into a Jβ2.7/Vδ2 CSJ target (Figure 8A). The breakpoints consisted of a perfect SJ on the right arm of the insertion; on the left arm, a junction compatible with a ΨHJ was found, displaying a deletion of three nucleotides on one side of the joint, a deletion of one nucleotide on the other side, and one N nucleotide addition; in the second case, the same ~900-bp Jδ1-Dδ2 fragment was observed inserted into a Dβ1/Vδ2 CSJ target (Figure 8B). Similarly, one of the breakpoints displayed a perfect SJ, and the other breakpoint consisted of an SJ with 2 N insertions. Considering the structure of the target, this last junction was ambiguous to assign as an SJ or a ΨHJ, and could have occurred through nick–nick, V(D)J, and/or RAG-mediated joining. Nevertheless, all junctions complied with the 12/23 rule and with features of RAG-mediated breaks, and it is therefore very likely that both cases represent in vivo examples of RAG-mediated reintegration of a Jδ1-Dδ2 ESJ. In the two cases, both ESJ reintegration and translocation events occurred, and two mechanisms could therefore account for their formation: either the *trans*-TCR translocations occurred first, and provided a *trans*-TCR SJ target for ESJ reintegration; or, alternatively, ESJ reintegration in a standard RSS target (e.g., Vδ2) could have occurred first, and provided a *trans*-TCR structure and/or breakpoints prone to V(D)J-mediated translocation. Intriguingly, the actual frequency of this double translocation/insertion event (2/120 = ~10^−2^ *trans*-recombination events) was not compatible with the expected combined frequency of each independent event: (*trans*-TCR translocation ~10^−4^–10^−6^ × reintegration ~10^−4^–10^−6^ = ~10^−8^–10^−12^). Furthermore, as the assay is limited to PCR-amplifiable sizes of the inserted fragment (such as the relatively short Jδ1-Dδ2 episome), the apparent rate of reintegration is probably vastly underestimated. This strongly suggests that the two events are linked, either because CSJs constitute preferential targets for ESJ reintegration, or because ESJ reintegration generates genomic instability leading to further chromosomal abnormalities; such abnormalities could consist of chromosomal translocations as detected here, or potentially more complex events which could not be detected in the present in vivo assay.

Altogether, these data demonstrate that in vivo, SJs located on excised ECs may be reintegrated into the genome through *trans*-V(D)J recombination using standard and cryptic RSS targets, and further suggest that this event is associated with additional genomic instability.

## Discussion

During B- and T-cell ontogeny, ECs are sequentially excised from the genome of lymphoid cells as a result of the hierarchically regulated D-to-J, V-to-DJ, and V-to-J rearrangement events. Such episomes are believed to be nonreplicative, but have nevertheless been shown to be surprisingly stable structures, persisting until diluted out by cell divisions [41,42]. Such episomes carry an ESJ comprised of two functional RSSs, each of which are susceptible to (re-)bind the RAG proteins when (re-)expressed at the various cell maturation steps until final downregulation [7,41,43]. On the other hand, *trans-*V(D)J recombination occurring between RSSs or cryptic RSSs located on distinct chromosomes is a relatively common event in developing lymphocytes [22,23,44], which has been shown to lead to recurrent oncogenic translocations [34]. Here we show both ex vivo and in vivo that ECs may be reintegrated into the genome through *trans*-V(D)J recombination occurring between the ESJ and an IG/TCR or one of the many cryptic RSS targets scattered in the genome.

### Molecular Mechanisms of ESJ Reintegration in Ex Vivo Assays

We have demonstrated in ex vivo assays that the efficiency of *trans*-V(D)J recombination of an ESJ with a RSS target is not quantitatively different from the *trans*-V(D)J recombination occurring between two RSSs. This is somehow unexpected, because its particular structure confers additional properties to the ESJ. ESJs are efficiently cleaved ex vivo and in vitro by the nick–nick mechanism, a symmetrical nick occurring 5′ of each RSS (simultaneously or sequentially) and generating two flush SEs ending with 3′ hydroxyl groups [7]. Remarkably, this process bypasses both the formation of a hairpin intermediate and the need of synapsis with another RSS. In the context of ongoing SJ recombination, one could think that efficient nick–nick opening of lone ESJs upon RAG binding might prevent the occurrence of synapsis with a RSS target, and thus considerably reduce the overall frequency of *trans*-V(D)J rearrangement. Our ex vivo data clearly argue against this assumption, and suggest that *trans*-V(D)J rearrangement is largely independent of nick–nick. This however does not preclude that ESJ opening by nick–nick might occasionally participate in the synapsis, and the recent in vivo demonstration of synapsis by capture [2] provides a plausible two-step scenario of the occurrence of nick–nick within a 12/23 synapse (Figure S4).

Incidentally, our data provides additional evidence that HJs can be formed ex vivo through both NHEJ-dependent and NHEJ-independent pathways (Figure S2). RAG-mediated joining has been initially proposed as an efficient alternative pathway of NHEJ-independent HJ formation [16–18]. However, conflicting data have been reported on the relevance of this pathway ex vivo and in vivo [15,25–27,45,46]. In particular, recent data indicated that at least ex vivo, RAG-mediated joining is seldom observed in presence of full-length RAGs, even in the absence of the concurrent NHEJ pathway [15,46]. We find here the presence of HJ with features of RAG-mediated joining in the absence, but not in the presence of Artemis, suggesting that at least ex vivo, HJ formation is a mixed process consisting of efficient RSS “swapping” and inefficient RAG-mediated joining. Interestingly, it is possible that the frequency of RAG-mediated joining would be favored in our assay, due to the participation of an ESJ. As mentioned above, nick–nick opening of the ESJ in the context of a 12/23 synaptic complex would generate two flush SEs ending with 3′ hydroxyl groups. While the two reactive RSSs might be sequestered by tight binding in the SE postcleavage complex (Figure S4), this additional free 3′ OH would provide an available substrate for hairpin attack, at least in absence of concurrent hairpin resolution by Artemis. Thus, nick–nick opening might participate in both *trans*-V(D)J recombination and RAG-mediated joining.

### In Vivo Reintegration of Excised ECs by the V(D)J Recombinase

Using WT and DE double-mutant mice, we could estimate that ESJ reintegration in authentic TCR RSS targets occurs in vivo in the broad range of 1 in 1,000,000 to 1 in 10,000 mouse thymocytes, depending on the integration site. In agreement with our results, recent data provide further evidence for the existence of V(D)J-mediated ESJ reintegration in vivo, and concur with the idea that it constitutes a significant source of genomic instability. Using a screen for *HPRT* mutants, Finette and colleagues recently identified a case in which a Vα/Jα episome was found inserted in the cryptic 23-RSS from *HPRT* intron 1 [39]. This finding is all the more remarkable given that this *HPRT* cryptic site has been shown to exhibit a very low recombinogenic potential in functional assays ([47], our unpublished data), and provides direct evidence that in vivo, human cryptic RSSs constitute efficient targets for ESJ reintegration. In the same line, using a double selection assay allowing for the recovery and quantification of excision/reinsertion events in a pre–B-cell line, Reddy et al. recently identified three cases of V(D)J-mediated reintegration [48]. In full agreement with our in vivo estimation, they evaluated a rate of one reintegration out of every 100,000 V(D)J recombinations. Considering the average number of V(D)J recombination per B or T cell, these data suggest that the daily lymphocyte output in human could be accompanied by as many as 5,000 ESJ reintegration events.

### A Potential Threat for Genomic Stability and Lymphoid Neoplasia

Authentic RSSs from IG/TCR must without doubt constitute preferential targets for such reintegration. However, there are an estimated 10 million functional cryptic sites dispersed throughout the human genome [34,47], some of them displaying much higher recombinogenic potential than the *HPRT* intron 1 cryptic RSS in which ESJ reintegration was found [29,37]. Most important, some of them are already known to provide recurrent targets for oncogenic (type 1) V(D)J-mediated translocations. In T-ALL, for example, the mistargeting of the RAGs towards such cryptic RSSs located in the vicinity of a silent proto-oncogene is a recurrent source of genomic instability and oncogenesis [34]. We demonstrate in our ex vivo assay that cryptic sites involved in oncogenic V(D)J-mediated translocations are also hotspots for ESJ recombination; it seems thus reasonable to think that in vivo, such cryptic RSSs might provide as efficient targets for ESJ reintegration than for chromosomal translocations. ESJ reintegration could lead to similar oncogenic activation/deregulation, either through the insertion of active immune regulatory elements (e.g., the TCRδ enhancer excised during ΔRec/ΨJα or Vα/Jα rearrangements [42,49], or the IGK enhancer excised during KDE/Ki rearrangements [12,50]) or through the disruption of locus silencing (e.g., the negative regulatory element upstream of *LMO2* [51,52], WA Dik; BN et al., unpublished data).

If ESJs are at least as efficient as any standard coding-segment RSS to undergo V(D)J recombination with a RSS target in *trans,* the two processes are nearly identical mechanistically, and the RSS/cryptic RSS targets are the same, why then has V(D)J-mediated oncogenic translocation been largely documented in the literature, but V(D)J-mediated reintegration never been reported up to now in lymphoid malignancies? Higher-order spatial genome organization is a contributing factor in the formation of recurrent translocations [53]. In contrast, increased mobility of the EC might favor interactions and recombination of ESJs, with targets located in parts of the genome generally segregated. Oncogenic targets might thus be distinct for reintegration and translocation. However, due to the multiplicity and size range (from <1 Kb to several Mb) of the excised episomes, as well as the diversity of insertion targets, reintegration events are very unlikely to be detected by routine analysis. Particularly, and in contrast to chromosomal translocations, episomal reintegration is invisible by standard karyotype analysis [54,55]. Few examples of identified class-switch recombination– and RAG-mediated episomal reintegration in lymphoid neoplasia provide proof of principle that episomal reintegration can indeed lead to cancer, and illustrate the complexity and unlikelihood of identifying such events without specifically designed screens [55–57]. For example, using complex DNA fiber-FISH and three-color interphase FISH techniques on samples from follicular lymphoma patients devoid of the hallmark t(14;18)(q32;q21) translocation, Vaandrager et al. identified two cases in which the *BCL2* gene was excised from 18q21 and inserted into the IGH locus at 14q32 [58]. The relatively high frequency (5%) of such events, discovered relatively recently by Vaandrager et al. out of an abundantly studied and characterized pathology, illustrate well the extent to which insertional events might be missed by routine cytogenetic and molecular analysis. Another possibility which could account for the rarity of cases of episomal reintegration observed in lymphoid neoplasia is that episomal reinsertions might generate unstable transitory structures in the genome, leading to additional aberration events and more complex genomic configuration. This possibility is supported by the unexpected high frequency of two in vivo cases observed in this study, combining ESJ reintegration and translocation events. Similar examples of genomic instability following reintegration of episomal structures have been previously documented. In mouse plasmacytoma, Kovalchuk and colleagues have shown that class-switch–mediated Eμ/Sμ episomal reintegration into c-MYC favors t(12;15) translocation [56]. Although the ground of such instability is not yet clear, an obvious possibility is that a fraction of the recombination events might lead to incomplete insertion in vivo. Alternatively, the reinserted episomes might in vivo retain their initial “open” chromatinized structures, and provide preferential accessible targets for the recombination machinery. The V(D)J-mediated instability of reinserted ESJs would be especially relevant in neoplastic cells such as T-ALLs, in which arrest of differentiation at early stages of T-cell development leads to sustained RAG levels.

Gene profiling studies have shown that a substantial fraction of the T-ALL cases display oncogenic activation in absence of detectable chromosomal alterations [59], suggesting the presence of alternative pathways of oncogenesis. Some of them have indeed been recently discovered, and involve episomal structures [60]. Considering the large number of ESJs produced daily and the mechanistic similarities between ESJ reintegration and oncogenic translocations, our data suggest that reintegration of excised ECs by the V(D)J recombinase might also account for some of these cases, and constitute an additional potent source of genomic instability. Definitive answer to this open question will however await large-scale screens of human lymphoid cancer samples with specifically adapted strategies.

## Materials and Methods

### Ex vivo *trans*-recombination substrate assay.

Recombination substrates were derived from a series previously described [29], and the constructs are summarized in Figure S5. The various gene segments containing the regions to recombine were amplified from human DNA with appropriate tailed primers and cloned in the recombination substrate using unique restriction sites (Mlu1, Sac2, Not1). The (Ki/Jκ3) ESJ was cloned (Not1/BamH1) in the pPCR-scriptAmp vector (Stratagene, http://www.stratagene.com). Some constructs were flanked by “tag” sequences, which were previously tested to be devoid of functional cryptic RSSs, and in which specific PCR primers were designed. NIH 3T3 Swiss mouse fibroblasts, and human Artemis-deficient GUETEL or Artemis-complemented GUETEL-A cell lines [30] (generously supplied by J.-P. de Villartay) were cultured in standard conditions (DMEM/10% FCS). Cells (2 × 10^6^) were transfected with 3 μg of each recombination substrate, 2 μg pEBB-RAG1, 2 μg pEBB-RAG2 expression vectors (generously supplied by C. Roman and S. Cherry), and 2 μg pCDNA3TdT expression vector (*TdT* cDNA from the pTDT expression vector [a generous gift from N. Doyen] recloned into pcDNA3 [Invitrogen, http://www.invitrogen.com]) using Superfect (Qiagen, http://www.qiagen.com) according to the instructions recommended by the manufacturer. Transfected cells were transferred to 10 ml DMEM supplemented with 10% FCS and cultured for 48 h. Cells were subsequently trypsinized, and plasmids recovered by alkaline lysis and phenol-chloroform extraction as previously described [29]. To test the possibility that *trans-*rearrangements could occur through a first step of homologous recombination between the identical core regions of the donor/acceptor plasmids, the *trans*-recombination assay was also performed with “coreless” excised linear fragments carrying the RSS target, and gave rise to similar results (Figure S6). Although we cannot formally exclude the possibility that when present, stretches of homology can facilitate the recombination process both ex vivo and in vivo, our data suggests that recombination efficiently occurs through direct *trans*-V(D)J synapsis as previously assumed for the assay [20].

### PCR/sequencing.

Breakpoints were PCR amplified from 1 μl (1/20) harvested bulk DNA with appropriate primers (summarized in Table S1) in the following conditions: 4 min at 94°C for 25 cycles (30 s at 94°C, 30 s at 64°C, and 30 s at 72°C), and 7 min at 72°C. Secondary double-nested PCRs were performed in the same conditions, and the amplification products cloned and sequenced as previously described [29].

### Semiquantitative PCR/PE assays.

Breakpoints were amplified in a single round PCR from 1 μl (1/20) harvested bulk DNA with appropriate primers (summarized in Table S2) in the same conditions as above, and otherwise undetectable amplification products were revealed by PE. PE is a sensitive alternative to Southern blot consisting of several cycles of DNA polymerization extending from a labeled primer until the end of a matrix DNA fragment (here a PCR product). This generates linear (nonexponential) accumulation of labeled fragments of specific size. PE assays were performed on 1.5 μl, 1.0 μl, and 0.75 μl of primary PCR using a nested IR800-labeled primer in the following conditions: 5 min at 95°C for 20 cycles (30 s at 95°C, 15 s at 60°C and 1 min at 70°C), and 1 min at 70°C with the EXCEL II kit (Epicentre Biotechnologies, http://epicentre.com), using an equal amount of all dNTPs and omitting ddNTPs. A portion (1.2 μl) of the reaction was used for running on a Li-COR 4200 DNA sequencer (http://www.licor.com). A sequencing reaction was performed in parallel on the appropriate unrecombined purified plasmid, using the same IR800-labeled primer and in the same reaction conditions (at the exception of the dNTP/ddNTP mix), and was run on the same gel, providing both a precise to-the-base size marker and a positive control for the reaction. Semiquantitative conditions were calibrated on serial dilutions of bulk DNA and of PCR amplifications (Figure S7A–S7C). Furthermore, to exclude possible bias due to difference in the efficiency of the PCR primer combinations used in the semiquantitative experiments, each comparison using different sets of primers was calibrated (Figure S7D–S7E). All PCR/PE assays were performed on at least four independent transfections with similar results.

### Flow cytometry and cell-cycle analysis.

Thymocyte preparation and cell sorting were performed as described previously [61]. Phycoerythrin-conjugated mAb against TCRβ (H57–597), purchased from BD PharMingen (http://www.bdbiosciences.com), was used for cell sorting of TCRβ^+^ thymocytes. The sorting windows were defined in such a way that only cells expressing high levels of TCRβ were purified.

### In vivo fluctuation PCR assay.

The principle of the assay has been previously described [29]. Detection of rare events by sensitive double-nested PCR gives rise to fluctuation in target amplification, depending on the presence or not of the event in the aliquot taken from the sample. Junctions were PCR amplified from multiple replicates of 2 μg DNA (or 50 ng for positive replicates out of the fluctuation range) isolated either from total thymocytes of WT or Eβ^−/−^ mice [40], or from TCRαβ^+^ sorted thymocytes from WT or DE double-mutant mice, using appropriate primers (see list in Protocol S1), and in the following conditions: 4 min at 94°C for 30 cycles (30 s at 94°C, 30 s at 64°C, and 30 s at 72°C), and 7 min at 72°C. Secondary double-nested PCRs were performed on 1 μl of each primary PCR replicate in the same conditions, and amplification products were cloned and sequenced as previously described [29]. Frequencies were calculated using Poisson assumption as previously described [62].

## Supporting Information

Figure S1 Ex Vivo *trans*-V(D)J Recombination of ESJs Obeys the 12/23 Rule and Is Not Nick–Nick Dependent (A) SJ and ΨHJ/HJ sequences obtained with the complementary primer combinations (1 + 3) and (2 + 4) (see Figure 2A) corresponding to a 12/12 synapsis.
(B) Ex vivo *trans*-V(D)J recombination of ESJs made of two 12-RSSs (Dβ1/Dδ1) or made of two 23-RSSs (Dβ1/Dδ1) in the context of a 12-RSS target (Jβ2.7). Constructs and primers are indicated in Figure S5.
(C) Ex vivo *trans*-V(D)J recombination of nonamerless ESJs (indicated as Δ9) in the context of a 12-RSS target. See Figure S5 for construct and primer details.
(3.7 MB PDF)

Figure S2 Three Possible Pathways of a RAG-Mediated Hybrid Junction Resulting from Ongoing Recombination of an ESJ in *trans*
(Left) ESJ opens by standard RAG-mediated nick and *trans*-esterification in the context of a normal 12/23 synapsis; despite its ability to bind the RAGs, the bystander 12-RSS of the ESJ behaves as a coding segment, and undergoes hairpin formation. Artemis-dependent hairpin resolution, further processing, and NHEJ-dependent repair leads to a ΨHJ displaying processing on both sides of the joint; nevertheless, processing may be more limited at the RSS side, due to protection conferred by RAG binding.
(Middle) SJ opening by nick–nick in the context of a standard 12/23 synapse (see Figure S4 for details) provides an ESJ 12-RSS with a free 3′ OH. As an alternative, a rare 12/12 synapsis might also provide an ESJ 12-RSS with a free 3′ OH. In an Artemis-dependent pathway, hairpins are resolved by Artemis, processing occurs, and intermediate products are mistakenly repaired by RSS “swapping,” leading to HJ formation. Because the ESJ 12-RSS did not go through a hairpin formation, and is bound to the RAGs, limited processing occurs at the RSS side.
(Right) In the Artemis-independent RAG-mediated joining pathway, a direct attack of the ESJ 12-RSS free 3′ OH into the hairpinned CE bypasses the hairpin resolution step, and results in the generation of a class of HJ displaying a full-size RSS joined to a coding sequence with limited processing (depending on the position of the attack in the hairpin). In sequences from Artemis-proficient cells, the virtual absence of joints with features of RAG-mediated joining, the minor representation of products from 12/12 synapsis, and the presence of nucleotide deletion/addition at both sides in most of the joints, indicate that in the vast majority of cases, ESJs opened by standard RAG-mediated nick and *trans*-esterification in the context of a normal 12/23 synapsis.
White triangles, 12-RSS; black triangles, 23-RSS. Dented lines represent nucleotide processing; Δ, nucleotide deletion; P, P nucleotide addition; N, N nucleotide addition. For clarity, the generation of only one hybrid junction is represented.
(351 KB PDF)

Figure S3 In Vivo Detection of *trans*-TCR ΨHJs in Mouse Thymocytes Top lanes depict germline sequences (heptamers, spacers, and nonamers are specified; heptamers and nonamers are underlined). Upper cases represent coding-segments. Lower cases represent (bystander) RSSs. Recombined clones are depicted underneath with homology to the germline sequences indicated by vertical lines. A schematic representation of the PCR assay used for the detection of Jβ2.7/Dδ(1/2) and Jβ2.7/Jδ2) ΨHJs is depicted. PCR primers used are indicated; for the Jβ2.7/Dδ combinations, some cases were ambiguous because the sequences could correspond either to ΨHJs with large deletions or to processed SJs (sequences marked SJ/ΨHJ). Nevertheless, at least half of the cases could be unambiguously assigned to a ΨHJ due to the presence of remaining nucleotides from the Dδ coding sequence. Italics indicate potential P nucleotides; bold type, N nucleotides. Nucleotides in parenthesis are ambiguous and could be assigned to either side of the joint.
(1.4 MB PDF)

Figure S4 Hypothetical Two-Step Scenario of the Occurrence of Nick–Nick within a 12/23 Synapsis In a first step, both the target 12-RSSs and the ESJ 12-RSSs would independently undergo a RAG-specific nick; in a second step, the RAG-associated prenicked 12-RSS target would capture the 23-RSSs of the prenicked ESJ, forming a synaptic complex and initiating the second, symmetrical nick at the ESJ, eventually resulting to its opening in absence of transesterification. Although flush ESJ 12-RSSs could also be provided by rare 12/12 synapses, the two-step nick–nick process provides a potential pathway by which HJs could be generated in the context of a 12/23 synapse (Figure S2). Ellipses represent RAG-1/2, regardless of stochiometry.
(1.2 MB PDF)

Figure S5 Schematic Representation of the Recombination Cassettes (to Scale) Inserted in the Donor (ESJ, RSS) and Target (RSS) Recombination Substrates Cassettes and tags are delimited by lozenges, and PCR/PE primers are indicated. Broken line in the (Ki/Jκ3) ESJ represents core vector sequence. M, Mlu1; N, Not1; Sc, Sac2.
(1.5 MB PDF)

Figure S6 Ex Vivo *trans*-V(D)J Recombination Assay with Coreless Target Plasmid The 12-RSS target is located on a linear fragment excised from the plasmid core, and is devoid of large regions of homology with the ESJ plasmid. See also legend to Figures 2 and 3 for PCR/PE assay and breakpoint sequences.
(1.4 MB PDF)

Figure S7 Calibration of the PCR/PE Assay The PCR/PE assay was tested by performing PCR amplification on serial dilutions of bulk DNA harvested from transfections.
(A) A portion (10 μl) of the reaction was loaded on ethidium bromide–stained 1% agarose gel (M, 100-bp marker). The expected band (319 bp) is not detectable at any dilution.
(B) A portion (1 μl) of the PCR was then used for the PE assay, in the same conditions as described in Materials and Methods. The PCR/PE assay for the (Jδ1/Dδ3) ESJ/Jβ2.7 T1 transfection is shown, and illustrates the dynamic range of the assay. Bulk DNA (1 μl) was subsequently used as the standard condition for semiquantitative PCR.
(C) PE assay conditions were also tested on serial dilutions of PCR amplification products performed on 1 μl harvested bulk DNA. Three dilutions (1.5 μl, 1.0 μl, 0.75 μl) were used as the standard condition for the PE assay.
(D) Primer calibrations. Triplex PCR amplifications were performed in the same conditions as the primary PCR described in Materials and Methods, using a 1:1:1 mix of the indicated primers, and on serial dilutions of a 1:1 mix of plasmid DNA constructs containing the appropriate tags (indicated in brackets); each primer couple amplified its specific target with similar sensitivity, indicating that there is no large bias in the PCR detection of the rearrangements due to the use of distinct primers.
(E) Similarly, the efficiencies of the various labeled PE primers were also compared on serial dilutions from primary PCR. PE assays were performed in the same conditions as described in Materials and Methods, using the indicated labeled primers (*), on serial dilutions of the last dilution point (0.25 pg) of the corresponding triplex PCR. Again, primer comparison shows a similar sensitivity, indicating that there is no large bias in the PE detection of the rearrangements.
(4.4 MB PDF)

Protocol S1 List of PCR Primers (29 KB DOC)

Table S1 Primer Combinations in Ex Vivo Cloning Experiments (17 KB XLS)

Table S2 Primer Combinations in Ex Vivo Semiquantitative Experiments (19 KB XLS)

## Abbreviations

CSJ: chromosomal SJ
CE: coding end
CJ: coding joint
EC: episomal circles
ESJ: episomal signal joint
HJ: hybrid joint
IG: immunoglobulin
NHEJ: nonhomologous end-joining
PE: primer extension
ΨHJ: pseudo-HJ
RSS: recombination signal sequence
SE: signal end
SJ: signal joint
T-ALL: T-cell acute lymphoblastic leukemia
TCR: T-cell receptor
TdT: terminal deoxynucleotidyl transferase
