# Supplementary figures and images for: In Vivo Reinsertion of Excised Episomes by the V(D)J Recombinase: A Potential Threat to Genomic Stability
Source: PLoS Biol. 2007 Feb 13;5(3):e43. doi: 10.1371/journal.pbio.0050043 (PMC1820826; doi:10.1371/journal.pbio.0050043)

A

$\Psi$ HJ & HJ (2+4)

SJ (1+3)

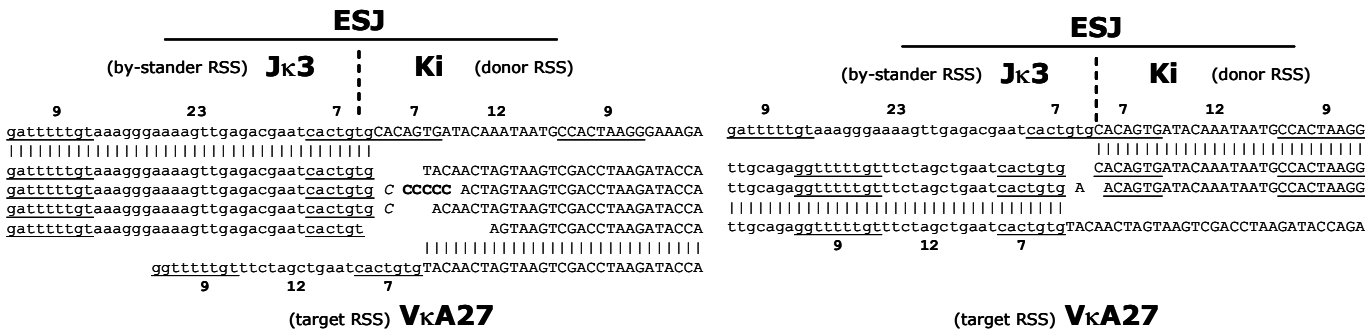

B

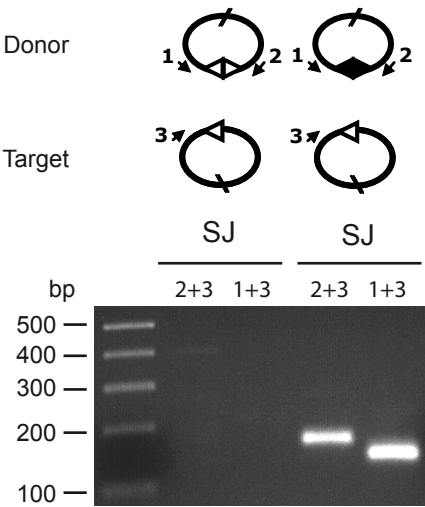

C

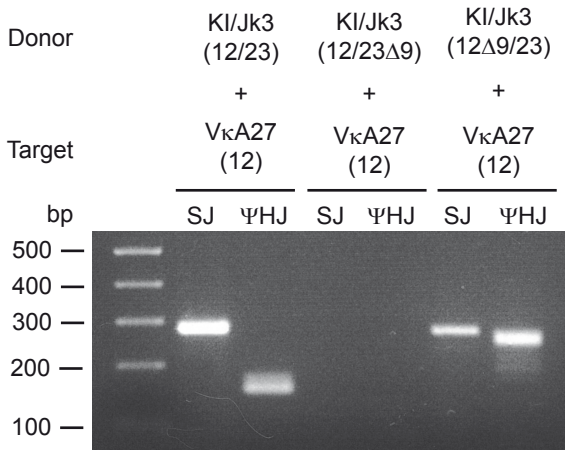

Supplement: Figure S1 — (A) SJ and ΨHJ/HJ sequences obtained with the complementary primer combinations (1 + 3) and (2 + 4) (see Figure 2A) corresponding to a 12/12 synapsis. (B) Ex vivo trans-V(D)J recombination of ESJs made of two 12-RSSs (Dβ1/Dδ1) or made of two 23-RSSs (Dβ1/Dδ1) in the context of a 12-RSS target (Jβ2.7). Constructs and primers are indicated in Figure S5. (C) Ex vivo trans-V(D)J recombination of nonamerless ESJs (indicated as Δ9) in the context of a 12-RSS target. See Figure S5 for construct and primer details. (3.7 MB PDF) [file pbio.0050043.sg001.pdf]

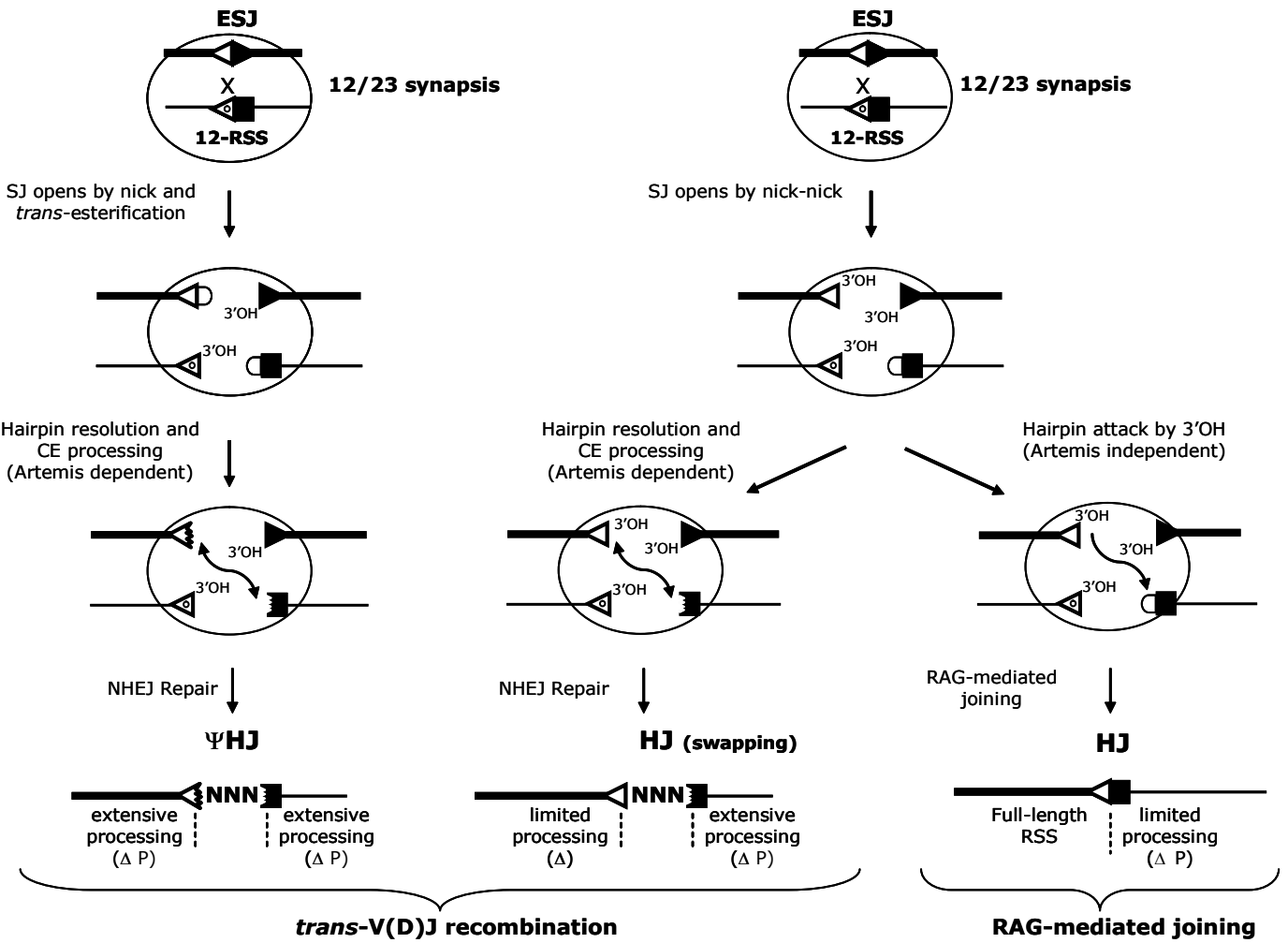

Supplement: Figure S2 — (Left) ESJ opens by standard RAG-mediated nick and trans-esterification in the context of a normal 12/23 synapsis; despite its ability to bind the RAGs, the bystander 12-RSS of the ESJ behaves as a coding segment, and undergoes hairpin formation. Artemis-dependent hairpin resolution, further processing, and NHEJ-dependent repair leads to a ΨHJ displaying processing on both sides of the joint; nevertheless, processing may be more limited at the RSS side, due to protection conferred by RAG binding. (Middle) SJ opening by nick–nick in the context of a standard 12/23 synapse (see Figure S4 for details) provides an ESJ 12-RSS with a free 3′ OH. As an alternative, a rare 12/12 synapsis might also provide an ESJ 12-RSS with a free 3′ OH. In an Artemis-dependent pathway, hairpins are resolved by Artemis, processing occurs, and intermediate products are mistakenly repaired by RSS “swapping,” leading to HJ formation. Because the ESJ 12-RSS did not go through a hairpin formation, and is bound to the RAGs, limited processing occurs at the RSS side. (Right) In the Artemis-independent RAG-mediated joining pathway, a direct attack of the ESJ 12-RSS free 3′ OH into the hairpinned CE bypasses the hairpin resolution step, and results in the generation of a class of HJ displaying a full-size RSS joined to a coding sequence with limited processing (depending on the position of the attack in the hairpin). In sequences from Artemis-proficient cells, the virtual absence of joints with features of RAG-mediated joining, the minor representation of products from 12/12 synapsis, and the presence of nucleotide deletion/addition at both sides in most of the joints, indicate that in the vast majority of cases, ESJs opened by standard RAG-mediated nick and trans-esterification in the context of a normal 12/23 synapsis. White triangles, 12-RSS; black triangles, 23-RSS. Dented lines represent nucleotide processing; Δ, nucleotide deletion; P, P nucleotide addition; N, N nucleotide addition. For clar [file pbio.0050043.sg002.pdf]

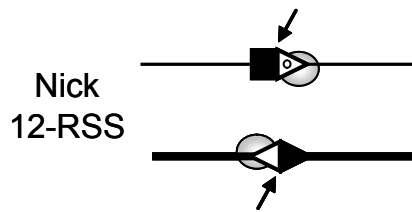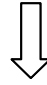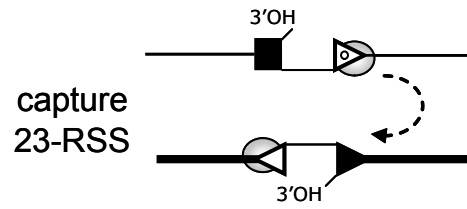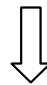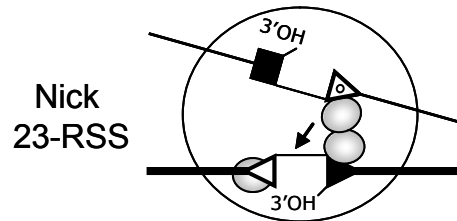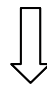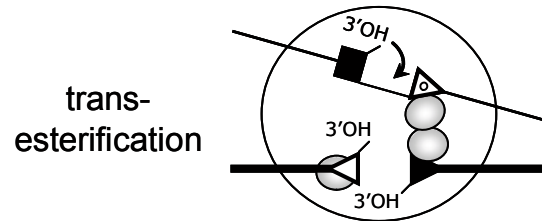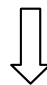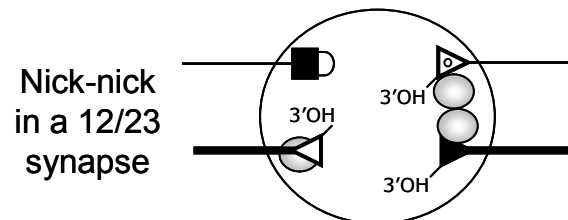

Supplement: Figure S4 — In a first step, both the target 12-RSSs and the ESJ 12-RSSs would independently undergo a RAG-specific nick; in a second step, the RAG-associated prenicked 12-RSS target would capture the 23-RSSs of the prenicked ESJ, forming a synaptic complex and initiating the second, symmetrical nick at the ESJ, eventually resulting to its opening in absence of transesterification. Although flush ESJ 12-RSSs could also be provided by rare 12/12 synapses, the two-step nick–nick process provides a potential pathway by which HJs could be generated in the context of a 12/23 synapse (Figure S2). Ellipses represent RAG-1/2, regardless of stochiometry. (1.2 MB PDF) [file pbio.0050043.sg004.pdf]

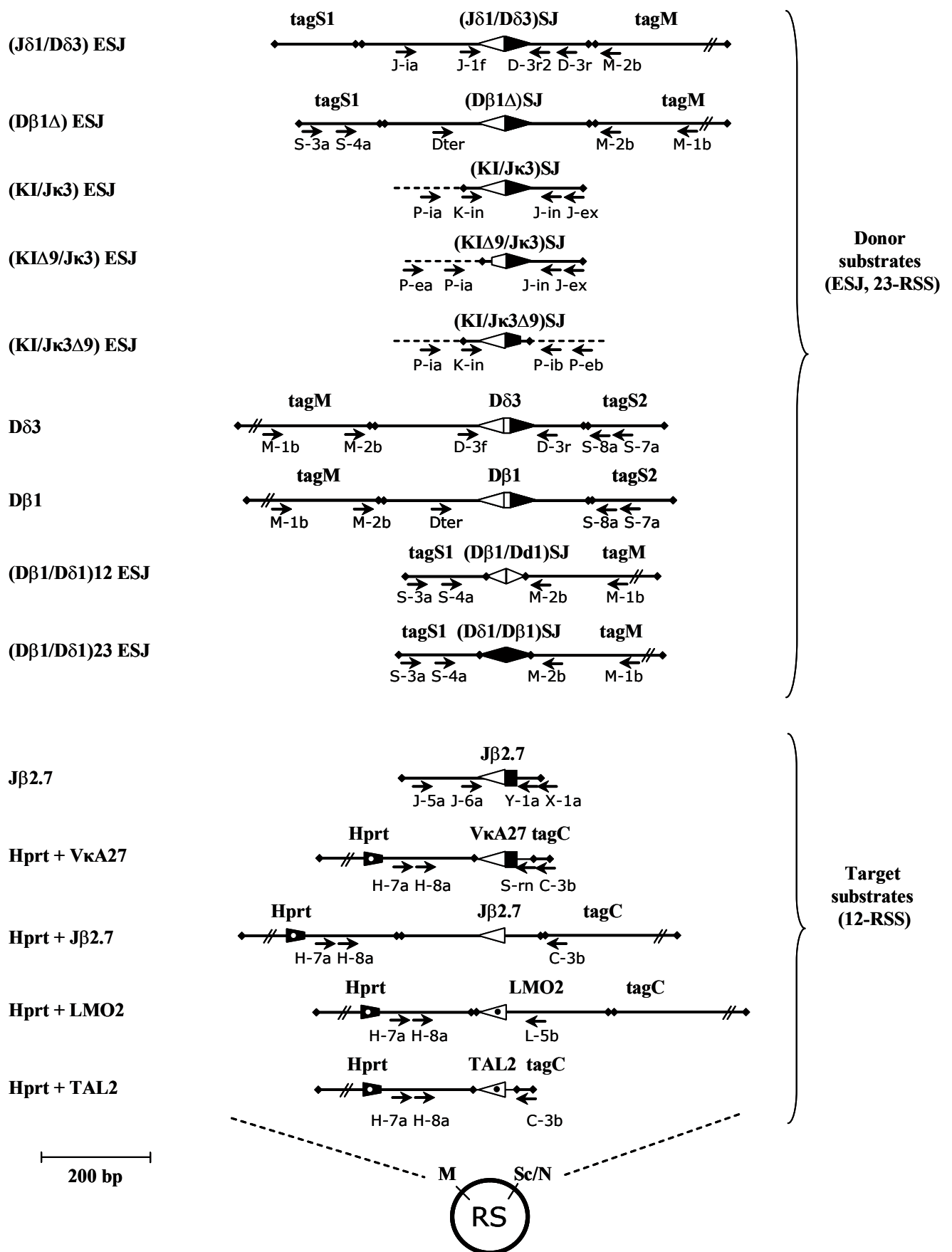

Supplement: Figure S5 — Cassettes and tags are delimited by lozenges, and PCR/PE primers are indicated. Broken line in the (Ki/Jκ3) ESJ represents core vector sequence. M, Mlu1; N, Not1; Sc, Sac2. (1.5 MB PDF) [file pbio.0050043.sg005.pdf]

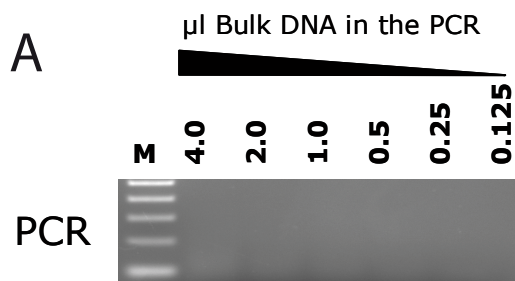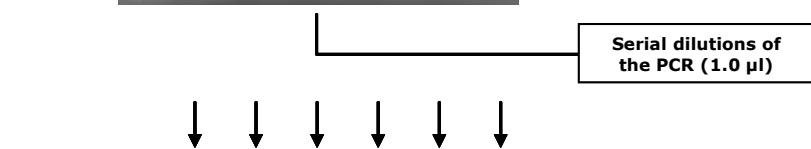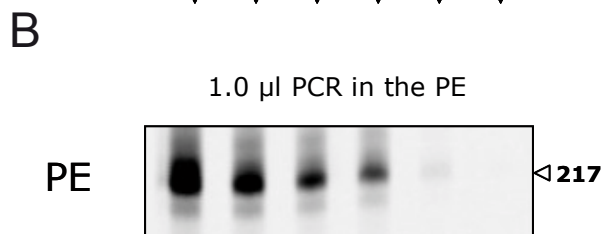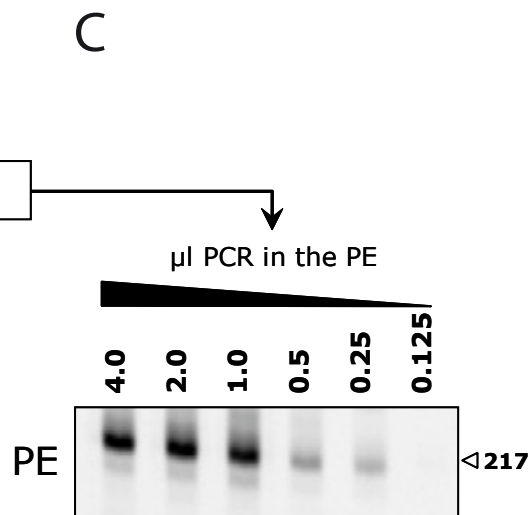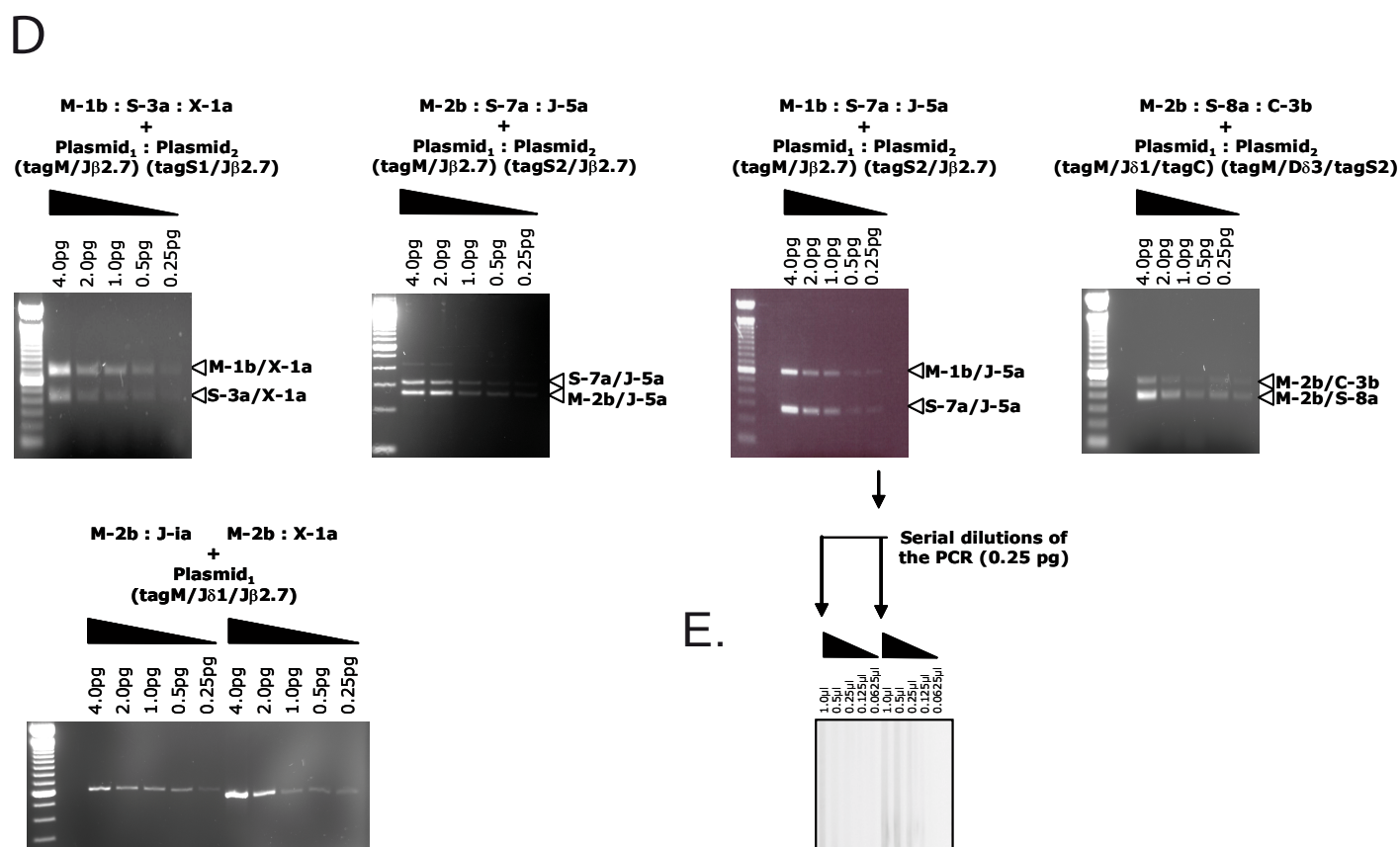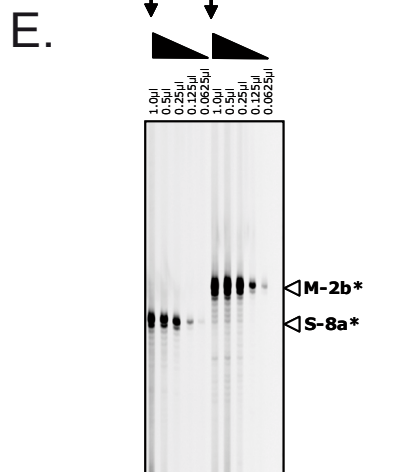

Supplement: Figure S7 — The PCR/PE assay was tested by performing PCR amplification on serial dilutions of bulk DNA harvested from transfections. (A) A portion (10 μl) of the reaction was loaded on ethidium bromide–stained 1% agarose gel (M, 100-bp marker). The expected band (319 bp) is not detectable at any dilution. (B) A portion (1 μl) of the PCR was then used for the PE assay, in the same conditions as described in Materials and Methods. The PCR/PE assay for the (Jδ1/Dδ3) ESJ/Jβ2.7 T1 transfection is shown, and illustrates the dynamic range of the assay. Bulk DNA (1 μl) was subsequently used as the standard condition for semiquantitative PCR. (C) PE assay conditions were also tested on serial dilutions of PCR amplification products performed on 1 μl harvested bulk DNA. Three dilutions (1.5 μl, 1.0 μl, 0.75 μl) were used as the standard condition for the PE assay. (D) Primer calibrations. Triplex PCR amplifications were performed in the same conditions as the primary PCR described in Materials and Methods, using a 1:1:1 mix of the indicated primers, and on serial dilutions of a 1:1 mix of plasmid DNA constructs containing the appropriate tags (indicated in brackets); each primer couple amplified its specific target with similar sensitivity, indicating that there is no large bias in the PCR detection of the rearrangements due to the use of distinct primers. (E) Similarly, the efficiencies of the various labeled PE primers were also compared on serial dilutions from primary PCR. PE assays were performed in the same conditions as described in Materials and Methods, using the indicated labeled primers (*), on serial dilutions of the last dilution point (0.25 pg) of the corresponding triplex PCR. Again, primer comparison shows a similar sensitivity, indicating that there is no large bias in the PE detection of the rearrangements. (4.4 MB PDF) [file pbio.0050043.sg007.pdf]
