# Supplementary material for: In Vivo Reinsertion of Excised Episomes by the V(D)J Recombinase: A Potential Threat to Genomic Stability
Source: PLoS Biol. 2007 Feb 13;5(3):e43. doi: 10.1371/journal.pbio.0050043 (PMC1820826; doi:10.1371/journal.pbio.0050043)
Supplement: Figure S3 — Top lanes depict germline sequences (heptamers, spacers, and nonamers are specified; heptamers and nonamers are underlined). Upper cases represent coding-segments. Lower cases represent (bystander) RSSs. Recombined clones are depicted underneath with homology to the germline sequences indicated by vertical lines. A schematic representation of the PCR assay used for the detection of Jβ2.7/Dδ(1/2) and Jβ2.7/Jδ2) ΨHJs is depicted. PCR primers used are indicated; for the Jβ2.7/Dδ combinations, some cases were ambiguous because the sequences could correspond either to ΨHJs with large deletions or to processed SJs (sequences marked SJ/ΨHJ). Nevertheless, at least half of the cases could be unambiguously assigned to a ΨHJ due to the presence of remaining nucleotides from the Dδ coding sequence. Italics indicate potential P nucleotides; bold type, N nucleotides. Nucleotides in parenthesis are ambiguous and could be assigned to either side of the joint. (1.4 MB PDF) [file pbio.0050043.sg003.pdf]

(by-stander) RSS **Jβ2.7** **Jδ2**

```
  9          12      7
ggtttgtgtgtggggttgagcctctgtg                                 CTCCTGGGACACCCGACAGATG
||||||||||||||||||||||||||||                                 ||||||||||||||||||||||
ggtttgtgtgtggggttgagcctctgt(g  c)                       (g  C)TCCTGGGACACCCGACAGATG  ΨHJ
ggtttgtgtgtggggttgagcctctgtg        GGATATCGGAGGGTCA   g  CTCCTGGGACACCCGACAGATG  ΨHJ
ggtttgtgtgtggggttgagcctctg                CA                TCCTGGGACACCCGACAGATG  ΨHJ
Ggtttgtgtgtggggttgagcct                                            ACACCCGACAGATG  ΨHJ
ggttttatgggggttgagc                     ATGTGT         g  CTCCTGGGACACCCGACAGATG  ΨHJ
ggtttgtgtgtggggttga                   AGGGGTCGGGGT     g  CTCCTGGGACACCCGACAGATG  ΨHJ
```

Jβ2.7/Jδ

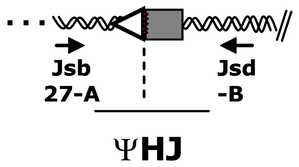

(by-stander) RSS **Jβ2.7** **Dδ1**

```
  9          12      7                                         7            23            9
ggtttgtgtgtggggttgagcctctgtg                    GTGGCATATCAcacaggttgaagtatattaaacctctgttcagaaacact
||||||||||||||||||||||||||||                    ||||||||||||||||||||||||||||||||||||||||||||||||||
ggtttgtgtgtggggttgagcctc              CTAT      GTGGCATATCAcacaggttgaagtatattaaacctctgttcagaaacact  ΨHJ
ggtttgtgtgtggggttgagcctctg             G        GTGGCATATCAcacaggttgaagtatattaaacctctgttcagaaacact  ΨHJ
ggtttgtgtgtggggttgagcctct              ATA       TGGCATATCAcacaggttgaagtatattaaacctctgttcagaaacact  ΨHJ
ggtttgtgtgtggggttg                     GG         GGCATATCAcacaggttgaagtatattaaacctctgttcagaaacact  ΨHJ
ggtttgtgtgtggggttgagcctctgtg cac       CC                CAcacaggttgaagtatattaaacctctgttcagaaacact  ΨHJ
ggtttgtgtgtggggttgagcctctgtg c         AAA               CAcacaggttgaagtatattaaacctctgttcagaaacact  ΨHJ
ggtttgtgtgtggggttgagcctctgtg c        GCTGG                 acaggttgaagtatattaaacctctgttcagaaacact  SJ/ΨHJ
ggtttgtgtgtggggttgagcctctgtg c                               caggttgaagtatattaaacctctgttcagaaacact  SJ/ΨHJ
ggtttgtgtgtggggttgagcctctg      GCCTATCCCCCCGCCGGAT          caggttgaagtatattaaacctctgttcagaaacact  SJ/ΨHJ
```

Jβ2.7/Dδ

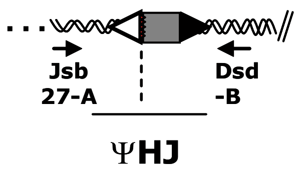

(by-stander) RSS **Jβ2.7** **Dδ2**

```
  9          12      7                                         7            23              9
ggtttgtgtgtggggttgagcctctgtg                ATCGGAGGGATACGAGcacagtgttgcaaaccccatagggacctgtacaaaaact
||||||||||||||||||||||||||||                ||||||||||||||||||||||||||||||||||||||||||||||||||||||||
ggtttgtgtgtggggttgagcctctgt         CCC  AT ATCGGAGGGATACGAGcacagtgttgcaaaccccatagggacctgtacaaaaact  ΨHJ
ggtttgtgtgtggggttgagcctctgtg      AGCCGGT      GGAGGGATACGAGcacagtgttgcaaaccccatagggacctgtacaaaaact  ΨHJ
ggtttgtgtgtggggttgagcctctgtg      GATGGA               ACGAGcacagtgttgcaaaccccatagggacctgtacaaaaact  ΨHJ
ggtttgtgtgtggggttgagcctc            AGT                    Gcacagtgttgcaaaccccatagggacctgtacaaaaact  ΨHJ
ggtttgtgtgtggggttgagcctctgtg                                cacagtgttgcaaaccccatagggacctgtacaaaaact  SJ/ΨHJ
ggtttgtgtgtggggttgagcctctgtg                                cacagtgttgcaaaccccatagggacctgtacaaaaact  SJ/ΨHJ
ggtttgtgtgtggggttgagcctct         ATACAG                      cagtgttgcaaaccccatagggacctgtacaaaaact  SJ/ΨHJ
ggtttgtgtgtggggttgagcct          ACTTGTCAG                    cagtgttgcaaaccccatagggacctgtacaaaaact  SJ/ΨHJ
```
