# Supplementary material for: In Vivo Reinsertion of Excised Episomes by the V(D)J Recombinase: A Potential Threat to Genomic Stability
Source: PLoS Biol. 2007 Feb 13;5(3):e43. doi: 10.1371/journal.pbio.0050043 (PMC1820826; doi:10.1371/journal.pbio.0050043)
Supplement: Figure S6 — The 12-RSS target is located on a linear fragment excised from the plasmid core, and is devoid of large regions of homology with the ESJ plasmid. See also legend to Figures 2 and 3 for PCR/PE assay and breakpoint sequences. (1.4 MB PDF) [file pbio.0050043.sg006.pdf]

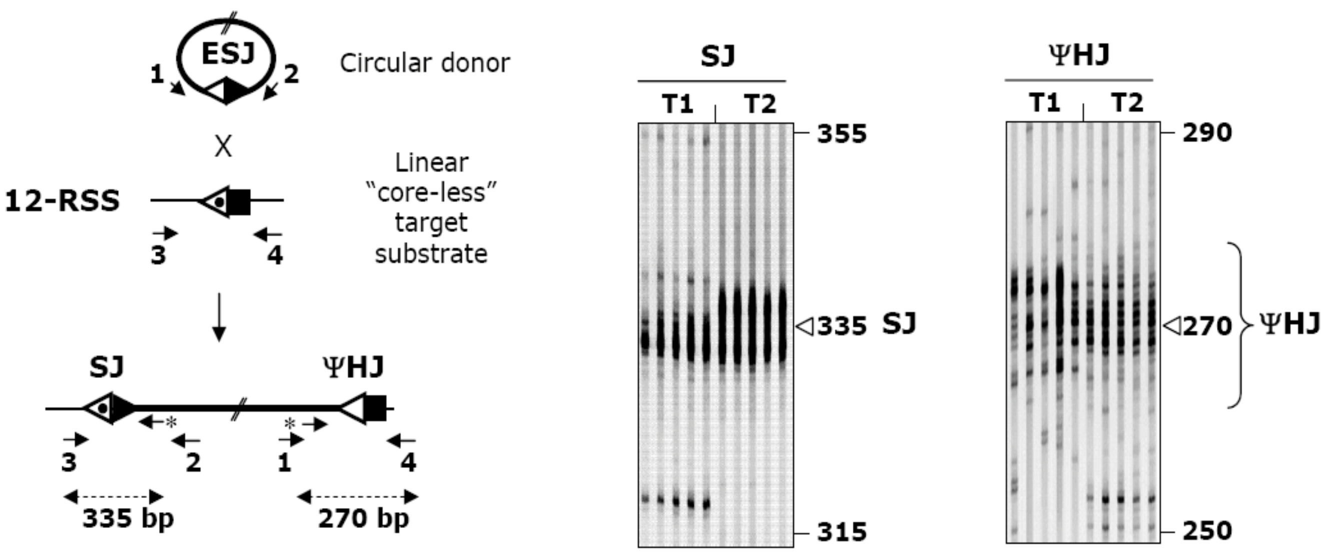

SJ

12-RSS

```
aattattgctgggtaagacaatactgtgTGTGTTACATAGCATTGGAACTTAGGAAAGCTCTTTCCTTGTGTTGT
||||||||||||||||||||||||||||
aattattgctgggtaagacaatactgtg        cacagtgctacaaaacctacagagacctgtacaaaaact
aattattgctgggtaagacaatactgtg        cacagtgctacaaaacctacagagacctgtacaaaaact
aattattgctgggtaagacaatactgtg  AGGG  cacagtgctacaaaacctacagagacctgtacaaaaact
                                    |||||||||||||||||||||||||||||||||||||||
AGCGCTGAGGTTTTTGGAACGTCCTCAAGTGCTGTGcacagtgctacaaaacctacagagacctgtacaaaaact
```

ESJ

ΨHJ

ESJ

```
GGTTTTTGGAACGTCCTCAAGTGCTGTGcacagtgctacaaaacctacagagacctgtacaaaaact
||||||||||||||||||||||||||||
GGTTTTTGGAACGTCCTCAAGTGCTGTG     G                GCATTGGAACTTAGGAA
GGTTTTTGGAACGTCCTCAAGTGCTGTG               TTACATAGCATTGGAACTTAGGAA
GGTTTTTGGAACGTCCTCAAGTGC               TGTGTTACATAGCATTGGAACTTAGGAA
                                       ||||||||||||||||||||||||||||
ccgtgcaccgaaattattgctgggtaagacaatactgtgTGTGTTACATAGCATTGGAACTTAGGAA
```

12-RSS
