# Supplementary material for: In Vivo Reinsertion of Excised Episomes by the V(D)J Recombinase: A Potential Threat to Genomic Stability
Source: PLoS Biol. 2007 Feb 13;5(3):e43. doi: 10.1371/journal.pbio.0050043 (PMC1820826; doi:10.1371/journal.pbio.0050043)
Supplement: Protocol S1 — (29 KB DOC) [file pbio.0050043.sd001.doc]

**Supplemental Material online**

*Oligonucleotides*

*Ex vivo Primers*

C-3b: 5’ GGAAGCAACCCACAAGTACGCG 3’

D-310: 5’ CCTTTGATCTTGCTGGAGCTTGACTTGG 3’

D-3f: 5’ ACATCTGGTTCTGGGGATTCTCAGGG 3’

D-3r: 5’ AGGGTGAGGATATCCCAGGGAAATGG 3’

D-3r2: 5’ GGAAATGGCACTTTTGCCCCTGCAG 3’

D-ter: 5’ TGGTGGTCTCTCCCAGGCTCTG 3’

H-7a: 5’ CAAGCCGAAATTCACATGTGCTAGAATCCAG 3’

H-8a: 5’ GCTGACCGATGCCCCAGGATATAG 3’

J-1f: 5’ AAAGCTGACGGGAAGAAGGTAGGTGG 3’

J-5a: 5’ GGCGGGATTCAGGTGGAAGG 3’

J-6a: 5’ GAGCTCGGGGAGCCTTAGAGG 3’

J-ex: 5’ GTTCCACAGGACGCTAGCTTGTGG 3’

J-ia: 5’ CATACTCCTCAGACAACAGCCTTCCG 3’

J-in: 5’ GAGGCTTGCTCACCTTTCAGGGAG 3’

K-in: 5’ CAGCTCAGCGCCCATTACGTTTCTG 3’

L-5b: 5’ GTACCCACTTTGCAGGGTTGTTGAGTGG 3’

M-1b: 5’ AGACTCAGGGCTTATCTCACCTTCTCAG 3’

M-2b: 5’ GTACAGATAACCCCCATATTCCACACCTG 3’

P-ia: 5’ TCCCAGTCACGACGTTGTAAAACGAC 3’

P-ea: 5’ GATGTGCTGCAAGGCGATTAAGTTGG 3’

P-ib: 5’ ATGCTTCCGGCTCGTATGTTGTGG 3’

P-ia: 5’ GTTAGCTCACTCATTAGGCACCCCAGG 3’

S-3a: 5’ GGTAAGTGGAGCTTTGTGCTCCTGG 3’

S-4a: 5’ GTGGGTAACATTCAAAGCCCTGTAGTGG 3’

S-7a: 5’ TGCGAGAATGCTATGCGTGTGAGTACAG 3’

S-8a: 5’ GGAGAAGGTTTCACAGAGGAAGTGACAG 3’

S-rn 5’ TACGCGGCCGCTCGATTGGCGCGACAAG 3’

X-1a: 5’ AATCACCGGCCGCATCTCACCTG 3’

Y-1a: 5’ ATCTCACCTGTGACCGTGAGCCTG 3’

*In vivo fluctuation PCR primers*

Dsb1-1A: 5’ GCATCTTACCACCACCTTGCACAATGG 3’ primary PCR

Dsb1-2A: 5’ GAGCAGCTTATCTGGTGGTTTCTTCCAG 3’secondary PCR

Dsb1-3B: 5’ GACCCAGGAGAAGAGTAGAGGACAG 3’ primary PCR

Dsb1-4B: 5’ GGGCCTTGGGACAGACAGAATGG 3’secondary PCR

Jsb27-1A: 5’ GCCTATGCGAGCTTCTTGGCAACTG 3’ primary PCR

Jsb27-2A: 5’ GGGAGGCTGAGTAGCAGGGAG 3’ secondary PCR

Jsb27-3B: 5’ CCCAGAAAGGGTGAAGTTGAGAGCTG 3’ primary PCR

Jsb27-4B: 5’ CCCTGGTCTACTCCAAACTACTCCAG 3’ secondary PCR

Vsd2-1A: 5’ CTCGTCTCCTTGGTGACATTACAGAAAGG 3’ primary PCR

Vsd2-2A: 5’ ATGCAGCAAAGTTCCCTGCAGATCCAAG 3’secondary PCR

Vsd2-4b: 5’ GTTAGTGAGGAAGGCAAACAGCAGGAG 3’ primary PCR

Vsd2-3b: 5’ GTAATGCTGAAGACACAGCCTCCCATTG 3’secondary PCR

Dsd1-3b: 5’ CTACAGCGCTCAATGGACTCTTTGCAG 3’ primary PCR

Dsd1-4b: 5’ CAGTGCTAAGACTTATACAGGGAGTCACAG 3’secondary PCR

Dsd2-1A: 5’ CTGGGGCTTCTCAGAGACACGTG 3’ primary PCR

Dsd2-2A: 5’ CACGTGATACAAAGCCCAGGGAAGG 3’secondary PCR

Dsd2-3b: 5’ CTGTGTTTACCTTCCATGGTGGCTGG 3’ primary PCR

Dsd2-4b: 5’ GGAGACGGTTCTTCACCCTGCAG 3’secondary PCR

Jsd1-1b: 5’ CCAGGCATCTTACTCAACACGACTGG 3’ primary PCR

Jsd1-2b: 5’ CTTCCAACCTCTTTAGGTAGAAGATCC 3’secondary PCR

Jsd2-1b: 5’ GCATCTTCACATCTGTCTCACTCTAACTCTG 3’ primary PCR

Jsd2-2b: 5’ GGCTCCACAAAGAGCTCTATGCCAG 3’secondary PCR
